# Controlling Ibrutinib’s Conformations about Its Heterobiaryl Axis to Increase BTK Selectivity

**DOI:** 10.1021/acsmedchemlett.2c00523

**Published:** 2023-02-14

**Authors:** Sean T. Toenjes, Bahar S. Heydari, Samuel T. Albright, Ramsey Hazin, Maria A. Ortiz, F. Javier Piedrafita, Jeffrey L. Gustafson

**Affiliations:** ^†^Department of Chemistry and Biochemistry and ^‡^Donald P. Shiley BioScience Center, San Diego State University, San Diego, California 92182-1030, United States

**Keywords:** Atropisomerism, BTK, Kinase, Target Selectivity, Oncology

## Abstract

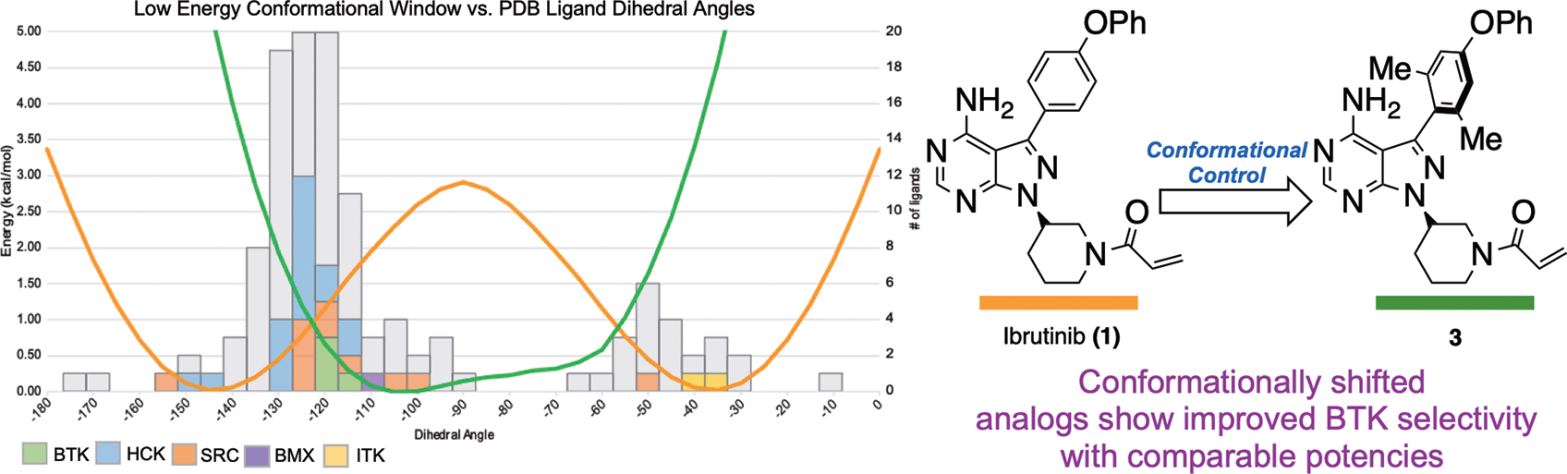

Ibrutinib is a covalent BTK inhibitor that is approved for several indications in oncology. Ibrutinib possesses significant off-target activities toward many kinases, often leading to adverse events in patients. While there have been robust medicinal chemistry efforts leading to more selective second-generation BTK inhibitors, there remains a need for new strategies to rapidly improve the selectivity of kinase inhibitors. An analysis of PDB data revealed that ibrutinib binds BTK in dihedral conformations that are orthogonal of ibrutinib’s predicted low energy conformational range. Synthesis of a series of analogues with ground state conformations shifted toward orthogonality led to the discovery of an analogue with two incorporated *ortho*-methyl groups that possessed markedly increased BTK selectivity. This work suggests that conformational control about a prospective atropisomeric axis represents a strategy to rapidly program a compound’s selectivity toward a given target.

Bruton’s tyrosine kinase (BTK) is a cytoplasmic Tec family kinase involved in controlling immune response through the B-cell receptor (BCR) signaling pathway.^1,2^ Aberrant BCR signaling can lead to autoimmune diseases such as rheumatoid arthritis (RA) and systemic lupus erythematosus (SLE) as well as B-cell malignancies such as mantle cell lymphoma (MCL) and chronic lymphocytic leukemia (CLL).^3,4^ A number of research groups have shown genetic knockdown or small molecule inhibition of BTK reduces cell growth in lymphoma cell lines and disease severity in RA and SLE mouse models.^5,6^ This has led to robust efforts leading to the FDA approval of the first-generation BTK inhibitor ibrutinib (**1**, Figure 1A) and, more recently, the second-generation BTK inhibitors acalabrutinib and zanubrutinib, for the treatment of patients with MCL (all 3 drugs) and CLL (ibrutinib and acalabrutinib).^6−10^ There are also several clinical BTK inhibitors such as rilzabrutinib, which is currently being evaluated for the treatment of thrombocytopenia and pemphigus among other diseases.^11,12^

**Figure 1:**
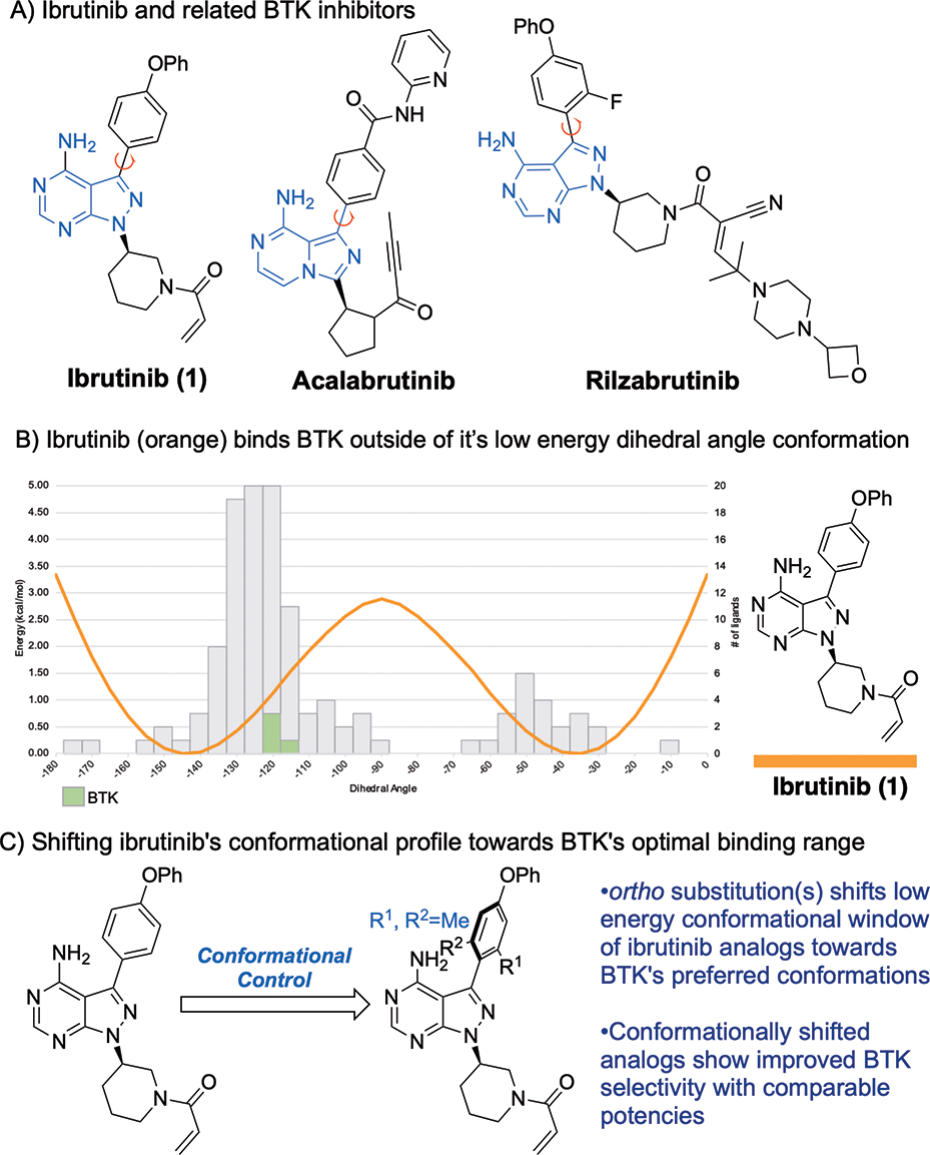
(A) BTK inhibitors with an atropisomeric or pro-atropisomeric aryl–aryl axis. (B) Predicted conformational energy profile of ibrutinib (orange) overlaid with the number of kinases bound to a PPY/PP scaffold in a given dihedral conformational range. (C) This work: conformational control of ibrutinib to increase selectivity.

Ibrutinib, along with the majority of BTK inhibitors, possess an electrophilic motif to covalently target BTK’s nucleophilic Cys481 residue located near the ATP-binding pocket. While ibrutinib possesses high affinity toward BTK, it lacks selectivity within the subset of kinases that possess a cysteine residue in a similar region.^6^ Off-target inhibition of these kinases, including ITK, TEC, BLK, EGFR, and JAK3, can lead to adverse events in patients such as rash, atrial fibrillation, and major hemorrhage.^13−15^ These side effects have largely prevented BTK inhibitors from being approved as therapeutics for autoimmune diseases. Because of this, immense efforts have focused on obtaining analogues of ibrutinib with improved selectivity by optimizing the 3-aryl group, the heterocyclic core scaffold or electrophilic handle.^16^

Atropisomerism is a conformational chirality that occurs when there is hindered rotation about a σ-bond that is exemplified by nonsymmetric aryl–aryl bonds.^17−20^ Atropisomers that are stereochemically unstable at 37 °C are quite prevalent in modern drug discovery with ∼^1^/_3_ of recent FDA approved small molecules (and ∼80% of FDA approved kinase inhibitors) possessing at least one potentially atropisomeric axis.^21,22^ Another 15% of FDA approved drugs possess what could be described as “pro-atropisomerism” wherein there is a plane of symmetry about the prospective atropisomeric axis. Ibrutinib, acalabrutinib, and the clinical BTK inhibitor tolebrutinib represent examples of pro-atropisomers, whereas rilzabrutinib likely exists as a stereochemically unstable atropisomer.

Recently, we have demonstrated that stereochemically stable atropisomeric analogues of pyrrolopyrimidines (PPYs) displayed improved selectivity compared to unstable atropisomeric PPYs, with each atropisomer inhibiting different kinases.^23,24^ To understand the origin of this selectivity, we “mapped” the conformations about this axis of 109 PPY or similar pyrazolopyrimidine (PP) ligands bound to different kinases (bar chart in Figure 1B), finding that the bulk of conformational space about the axis was sampled by different kinases. Comparing the bound conformations with the predicted conformational energy profiles (CEPs) for different PP/PPYs suggested that the major driver of selectivity was the preorganization of the aryl–aryl axis into a subset of conformations that were ideal for a given target but not for other kinases.^24^ This led to the hypothesis that preorganizing the CEPs of promiscuous unstable atropisomeric or pro-atropisomeric axes toward the preferred conformations of a target would allow for the “programming” of the scaffold’s selectivity toward that target. Beyond our work, there have been several recent examples of conformational control about a prospective atropisomeric axis to modulate the properties of biologically active small molecules, including Boehringer Ingelheim’s mutant EGFR inhibitors,^25^ Lorlatinib,^26^ and Grenning’s cannabinol derivatives.^27^ More recently, a team at Janssen has disclosed an atropisomeric BTK inhibitor where the configuration of the atropisomeric axis was crucial for activity.^28,29^

Herein we describe studies where we evaluated analogues of ibrutinib where the CEP of the 3-aryl–aryl axis is shifted to more orthogonal conformations by the addition of methyl groups adjacent to this axis. These simple perturbations led to large improvements in BTK selectivity with little effect on BTK potency. This suggests that optimizing the conformational profile about a potential atropisomeric or pro-atropisomeric axis can be an efficient approach to program the selectivity profile of a lead compound toward a desired target.

An analysis of our aforementioned “conformational map” for PPY and PP scaffolds (see Figure 1B) revealed that BTK bound its inhibitors (ibrutinib or close analogues) in conformations that were shifted approximately 30° orthogonal of the predicted “zero-point” energy of ibrutinib. This analysis suggests that to adopt its BTK binding conformation ibrutinib would be expected to pay a ∼1 kcal/mol or greater energetic penalty, which, with all things being equal, would result in an order of magnitude loss in potency. Furthermore, the conformational flexibility of ibrutinib may in part explain its promiscuity, as it can access myriad conformations that may be preferred by off-target kinases. For example, cocrystal structures of HCK, ITK, and SRC bound to PP/PPY ligands, have the aryl–aryl axes binding these targets in lower energy conformations than that of what is observed for the axis of ibrutinib bound to BTK (Figure 2).

**Figure 2:**
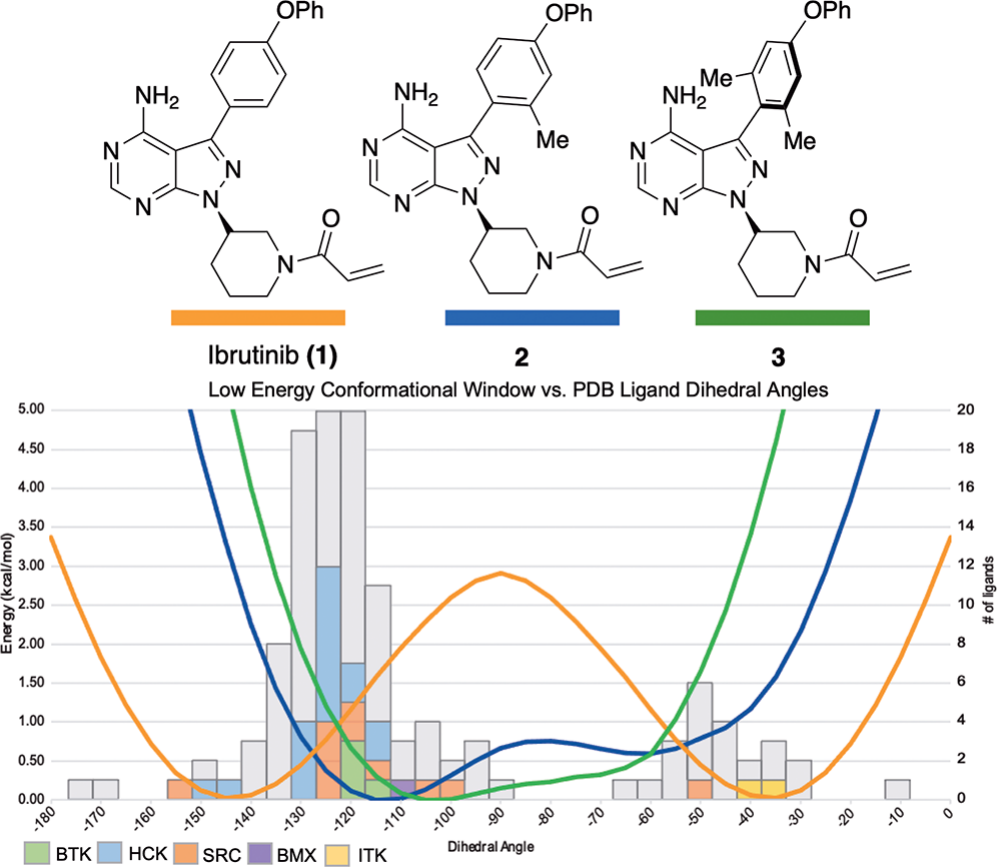
Predicted conformational energy profile of ibrutinib (**1**) (orange), **2** (blue), and **3** (green) overlaid with the number of kinases bound to a PPY/PP scaffold in a given dihedral conformational range obtained from known X-ray cocrystal structures deposited in the Protein Data Bank. For kinases that we obtained inhibition data for (Figure 3) are highlighted.

Based on these analyses, we undertook studies to evaluate whether preorganizing the aryl–aryl bond of ibrutinib toward BTK binding conformations could lead to increased target selectivity. To begin these studies, we calculated the CEP of several ibrutinib analogues with varying methyl substitutions adjacent to the aryl–aryl axis (displayed on a 0° to −180° scale) finding the addition of a single methyl group *ortho* to the axis as in **2**, is predicted to shift the “zero-point” energy roughly 30° toward orthogonality (−140° to −110^o^, orthogonal defined as −90°). Adding two *ortho* methyl groups, as in **3**, is predicted to shift the “zero-point” energy further orthogonal to now −100° with a narrower low energy conformational range. As analogues **2** and **3** possess conformational ranges that are closer to those preferred by BTK, we hypothesized that they would display improved selectivity toward BTK, as they would pay a smaller conformational energetic penalty to bind BTK and a potentially larger energetic penalty to bind off-targets that prefer less orthogonal conformations.

We synthesized **2** and **3** as shown in Scheme 1. To obtain **2**, we subjected the commercially available 3-iodo-1*H*-pyrazolo[3,4-*d*]pyrimidin-4-amine (**4**) to a Mitsunobu reaction with commercially available *N*-Boc-protected 3-hydroxypiperidine (**5**) to give **6**. Reacting isolated **6** with diaryl ether boronic ester **7** under standard Suzuki coupling conditions yielded **8**. Boc deprotection with TFA followed by addition of acryloyl chloride afforded **2** in good yield. A similar synthetic route toward **3** failed, likely because of the steric demand of the key Suzuki coupling. This led us to the strategy described in Scheme 1B, wherein we created the heterocycle–aryl bond *de novo*. We first subjected phenol **9** to a Chan-Lam coupling with phenyl boronic acid yielding diaryl ether **10**, which was reacted with activated Mg° and added into commercially available 4,6-dichloropyrimidine-5-carbaldehyde under inert atmosphere to afford an alcohol intermediate, which was subsequently oxidized using Dess–Martin periodinane to give ketone **11**. The ketone substrate was reacted with hydrazine monohydrate to yield PP **12**, which was then subjected to a Mitsunobu reaction with **5** to yield **13**. Amination with 2,4-dimethoxy benzyl amine yielded protected **14**, which was deprotected with TFA and acylated with acryloyl chloride to give **3**.

**Scheme 1:**
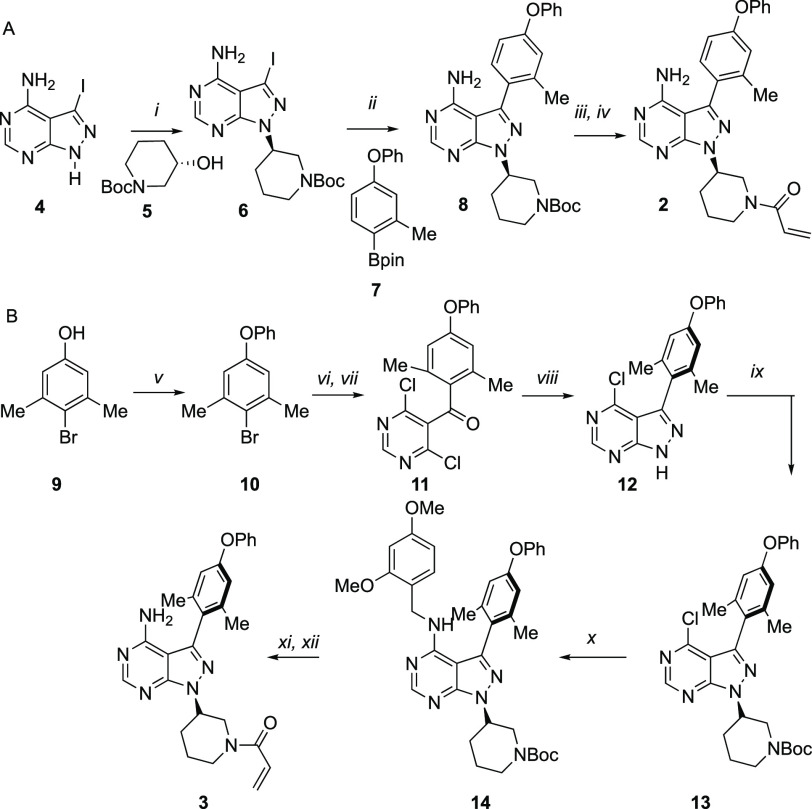
(i) *tert*-Butyl (*S*)-3-hydroxypiperidine-1-carboxylate, PPh_3_, DIAD, THF, 0 °C to 60 °C 18 h; (ii) Pd(PPh_3_)_4_, K_2_CO_3_, dioxane/H_2_O, 100 °C, 24 h; (iii) TFA, DCE, 60 °C, 2 h; (iv) acryloyl chloride, THF, 0 °C, 1 h; (v) PhB(OH)_2_, TEA, Cu(OAc)_2_, DCE, rt, 24 h; (vi) Mg^0^, Et_2_O, rt overnight followed by 4,6-dichloropyrimidine-5-carbaldehyde, −78°C to rt, 4 h; (vii) DMP, DCM, rt, 18 h; (viii) NH_2_–NH_2_, DIPEA, THF, 65 °C, 18 h; (ix) **5**, PPh_3_, DIAD, THF, 0 °C to 60 °C 18 h; (x) 2,4-dimethoxybenzylamine, Cs_2_CO_3_, dioxane, 80 °C, 12 h; (xi) TFA, DCE, 60 °C, 2 h; (xii) acryloyl chloride, THF, 0 °C, 1 h.

With **2** and **3** in hand, we first determined the *k*_inact_/*K*_*i*_^30^ of ibrutinib, **2**, and **3** (Figure 3). The *k*_inact_/*K*_*i*_ describes the efficiency of covalent bond formation of covalent inhibitors and is an important parameter used to rank covalent inhibitors in SAR studies. The reversible pre-equilibrium binding of the inhibitor to the enzyme is characterized by the binding constant, *K*_*i*_.^31^ The *k*_inact_ is a first-order rate constant which represents the maximum potential rate of covalent bond formation.^30^ We found ibrutinib to possess a *k*_inact_/*K*_*i*_ of 328 000 M^–1^ S^–1^, while **2** had a similar *k*_inact_/*K*_*i*_ of 280 000 M^–1^ S^–1^. Inhibitor **3** possessed an order of magnitude lower *k*_inact_/*K*_*i*_ of 31 000 M^–1^ S^–1^, which, while lower than ibrutinib and **2**, is in line with other covalent BTK inhibitors such as acalabrutinib and tirabrutinib.^31^

**Figure 3:**
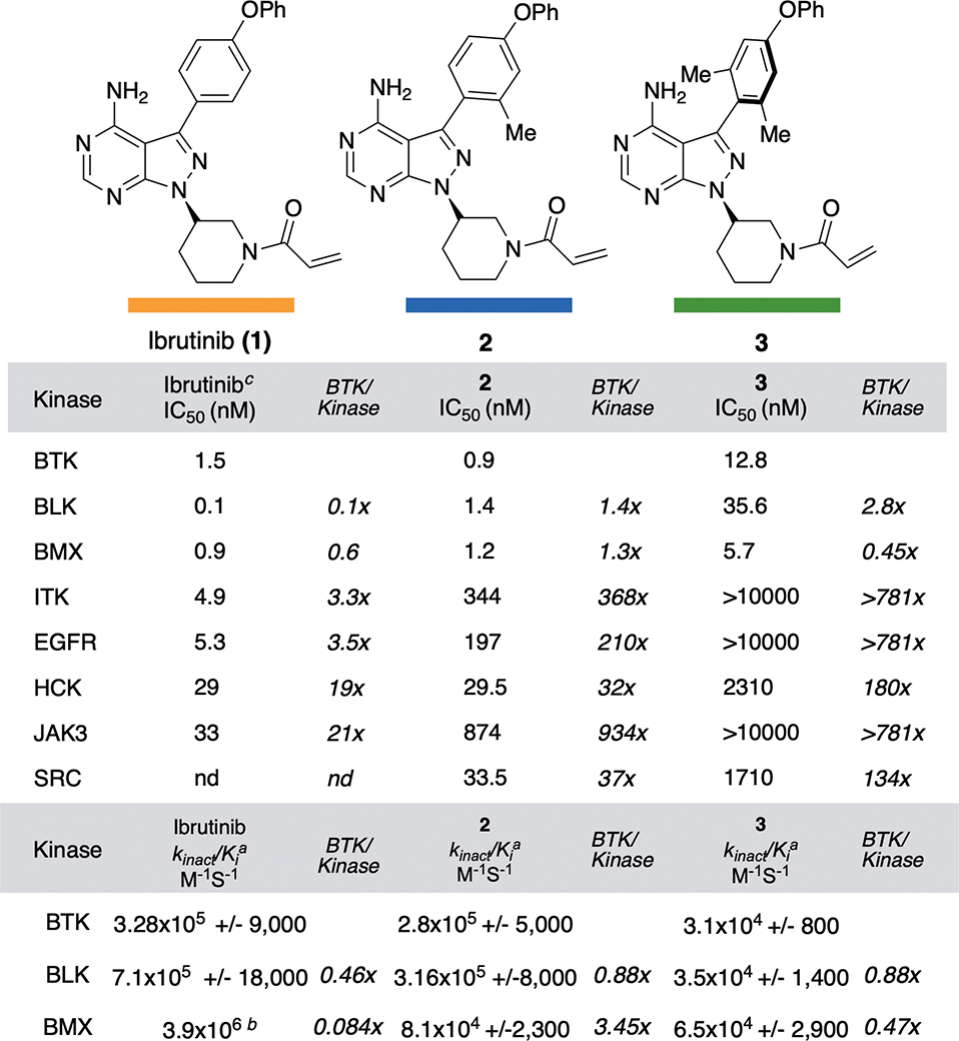
Kinase inhibition data for inhibitors ibrutinib (**1**), **2** and **3**. The *k*_inact_/*K*_*i*_ values were determined by AssayQuant Technologies (*a*), and taken from the literature (*b*).^31^ Ibrutinib (*c*) IC_50_ values are taken from literature.^7^ The IC_50_ values for **2** and **3** were determined in duplicate by ThermoFisher using the Z′-Lyte kinase assay. The data as well as error analysis are included in the Supporting Information.

We next evaluated these compounds across a panel of kinases known to be inhibited by ibrutinib. We chose to obtain IC_50_s by using a long incubation time (1 h) for these compounds across the panel, as recent work from Pfizer has suggested that IC_50_s with long incubation times yield meaningful data for covalent inhibitor SAR studies that largely agree with *k*_inact_/*K*_*i*_ values.^32^ In line with the *k*_inact_/*K*_*i*_ data, compound **2** suffered no loss in potency toward BTK compared to ibrutinib (0.9 nM), however, displayed a preference for BTK over BLK (1.4× compared to 0.1× for ibrutinib), ITK (368× compared to 3.3× for ibrutinib), EGFR (210× compared to 5.3× for ibrutinib), and JAK3 (934× compared to 21× for ibrutinib). As observed with the *k*_inact_/*K*_*i*_ data, compound **3** lost an order of magnitude in potency (12.8 nM), however, displayed no observable activities against ITK, EGFR, and JAK3, and significantly increased BTK activity over BLK (2.8× compared to 0.1× for ibrutinib) and HCK (180× compared to 19× for ibrutinib).

Compound **2** only displayed a moderate increase in BTK selectivity over BMX (1.3× compared to 0.6× for ibrutinib), while **3** displayed a slight preference for BMX over BTK. The observed BMX activity for compounds **2** and **3** is perhaps predicted by the conformational map in Figure 2 as BMX (purple) bound PP/PPY scaffolds in a near-orthogonal conformation that overlaps with the predicted low energy ranges of **2** and **3**.

As BLK and BMX represent the main off-target kinases for **2** and **3**, we obtained further *k*_inact_/*K*_*i*_ data (Figure 3). In the literature,^7^ it has previously been shown that ibrutinib exhibits higher *k*_inact_/*K*_*i*_ values for BLK (7.1 × 10^5^ M^–1^ S^–1^) and BMX (3.9 × 10^6^ M^–1^ S^–1^) compared to BTK (3.28 × 10^5^ M^–1^ S^–1^), essentially inactivating BMX an order of magnitude faster than BTK. Compound **2** showed improved BTK selectivity by this metric, with *k*_inact_/*K*_*i*_ values for BLK (3.2 × 10^5^ M^–1^ S^–1^) and BMX (8.1 × 10^4^ M^–1^ S^–1^) compared to BTK (2.8 × 10^5^ M^–1^ S^–1^), with **2** inactivating BTK 3–4-fold faster than BMX. Compound **3** also showed improved BTK selectivity compared to ibrutinib, albeit less so than **2** (*k*_inact_/*K*_*i*_ values for BLK (3.5 × 10^4^ M^–1^ S^–1^) and BMX (6.5 × 10^4^ M^–1^ S^–1^) compared to BTK (3.1 × 10^4^ M^–1^ S^–1^).

Intrigued by our observed selectivity, we profiled compounds **2** and **3** against ibrutinib’s top 50 targets^33^ at 1 μM (Figure 4). This data demonstrated a striking increase in kinase selectivity for **3** compared to ibrutinib and **2**. For example, ibrutinib inhibited each of these targets (50 kinases) at 50% of control or greater at 1 μM, with 26 of them inhibited to 10% of control. Compound **2** inhibited 19 of these kinases to 50% inhibition at 1 μM, with 17 inhibited to 10% of control activity. Compound **3** only inhibited 9 of these kinases (BTK, BLK, TXK, BMX, ERBB4, TEC, LCK, BRK, and FGR) to 50% of control at 1 μM, with only 6 (BTK, BLK, TXK, BMX, ERBB4, and TEC) being inhibited to 10% of control or greater at 1 μM.

**Figure 4:**
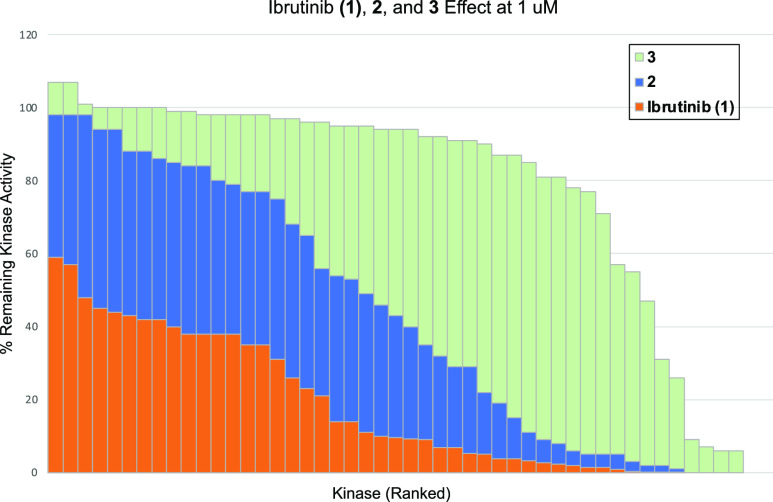
Single-point kinase profiling of ibrutinib (**1**), **2**, and **3** at 1 μM. Data for **2** and **3** was obtained from ThermoFisher using the SelectScreen kinase profiling assay. Data for ibrutinib is from the literature.^33^

Based on our predicted CEPs of **2** and **3**, we hypothesize that the increased selectivity of both compounds comes from the destabilization of more planar conformations that many of ibrutinib’s off-targets bind. The 10× loss of BTK potency for **3** is likely caused by **3**’s CEP “overshooting” BTK’s optimal conformational range, however, **3** still displays low nM potency, and the narrower CEP likely leads to the marked increases in selectivity that more than make up for the loss in potency.

For the most part, our observed inhibition data agrees with predictions that could be made from the “conformational map” in Figure 2. For example, compound **2** has significantly attenuated activity toward ITK compared to ibrutinib (344 nM vs 4.9 nM for ibrutinib), whereas **3** had no observable ITK activity. This trend overlays well with Figure 2, as the CEP predicts that **2** would have to adopt a pose ∼1.5 kcal/mol higher than its low energy range, which would result in over an order of magnitude loss in potency. Compound **3**’s CEP predicts a much higher energy conformation is needed to bind ITK (∼4 kcal/mol), which agrees with the lack of observed ITK inhibitory activity.

HCK and SRC kinases are known to exist in a multitude of different low energy conformations,^34,35^ which perhaps explains their diverse bound conformations in the dihedral angle map. Nonetheless, compound **3** possesses notably less HCK activity (2310 nM vs ∼29 nM for ibrutinib and **2**), which could be explained by the low energy conformations of the CEP of **3** encompassing fewer of HCK’s potential binding conformations. In our profiling experiment, we also observed that **3** inhibited SRC less than that of ibrutinib (57% percent of control for **3** vs 3% of control for ibrutinib), which could also be explained by the CEP of **3** encompassing fewer of SRC’s potential binding conformations.

We next evaluated these compounds for the inhibition of BTK phosphorylation in cells, finding compounds **2** and **3** to inhibit BTK autophosphorylation at similar levels to that of ibrutinib in Jeko-1 cells, a common cellular model for MCL (Figure 5A). We also evaluated cellular engagement of BTK by these compounds using standard NanoBRET assays, finding **2** and **3** to engage BTK with time dependent IC_50_s as expected for covalent inhibitors (Figure 5B). At 90 min post compound addition, we observed low nanomolar IC_50_s for both **2** (109 nM) and **3** (314 nM), which are slightly attenuated compared to that of ibrutinib (44 nM). This data largely tracks with our *k*_inact_/*K*_*i*_ data where compound **3** is a slower covalent deactivator than ibrutinib, nonetheless the marked increase in selectivity perhaps makes up for this, at least in terms of a potential chemical probe.

**Figure 5:**
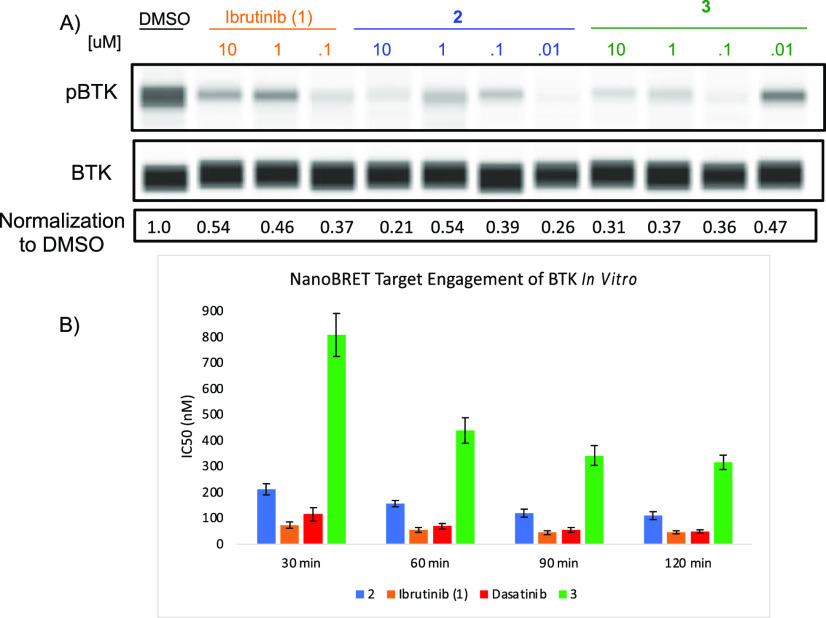
(A) Effect of ibrutinib (**1**), **2**, or **3** on the phosphorylation of BTK in Jeko-1 cells at 2 h. The pBTK/BTK ratio normalized to the DMSO control is shown in the bottom. (B) NanoBRET data for engagement of BTK by ibrutinib (**1**), **2**, or **3** in HEK293 cells transfected with the BTK-NanoLuc fusion vector. The data as well as error analysis are included in the Supporting Information.

In conclusion, during the course of analyzing the binding conformations of 3-arylpyrazolopyrimidines and related scaffolds, we observed the venerable BTK inhibitor ibrutinib bound BTK in conformations about a prospective atropisomeric axis that were shifted significantly orthogonal to its native low energy conformations. This led us to design and synthesize analogues of ibrutinib with orthogonal shifted conformations by simply adding methyl groups *ortho* to the aryl–aryl axis. These compounds, **2** and **3**, possessed significantly improved kinase selectivity compared to ibrutinib and displayed comparable activities toward BTK both *in vitro* and in BTK cellular models.

The studies described above are impactful as they demonstrate that the sampled “dihedral conformations” about a potential atropisomeric or pro-atropisomeric axis play a key role in the recognition of small molecules by proteins and that preorganizing a promiscuous small molecule into the preferred conformations of a target can “reprogram” the scaffold’s selectivity toward that target. While similar conformational effects have been previously discovered serendipitously and are often called “magic methyls”,^36^ this work perhaps provides a predictive data-based approach that can empower selectivity optimization. As prospective atropisomerism and pro-atropisomerism is ubiquitous in modern medicinal chemistry, this approach holds the potential to be a general strategy to obtain more selective compounds as leads toward safer and more efficacious therapies as well as more selective chemical probes.

## Supporting Information Available

^1^H NMR, ^13^C NMR, HPLC traces, experimental procedures, and characterization data for all new compounds (PDF)

## Abbreviations

BTK: Bruton’s tyrosine kinase
RA: rheumatoid arthritis
SLE: systemic lupus erythematosus
MCL: mantle cell lymphoma
CLL: chronic lymphocytic leukemia
PPY: pyrrolopyrimidine
PP: pyrazolopyrimidine
CEP: conformational energy profile
