# Supplementary material for: Controlling Ibrutinib’s Conformations about Its Heterobiaryl Axis to Increase BTK Selectivity
Source: ACS Med Chem Lett. 2023 Feb 14;14(3):305–11. doi: 10.1021/acsmedchemlett.2c00523 (PMC10009787; doi:10.1021/acsmedchemlett.2c00523)
Supplement: Supplementary file 1 — ml2c00523_si_001.pdf [file ml2c00523_si_001.pdf]

Controlling Ibrutinib's Conformations About its Heterobiaryl axis to Increase BTK Selectivity

Sean T. Toenjes[†], Bahar S. Heydari[†], Samuel T. Albright[†], Ramsey Hazin[†], Maria A. Ortiz[‡], F. Javier Piedrafita[‡], and Jeffrey L. Gustafson[*,†]

†Department of Chemistry and Biochemistry and ‡Donald P. Shiley BioScience Center, San Diego State University, San Diego, California, United States

Supporting Information

Table of Contents:

I. General Information……………………………………………………………………….3

II. Substrate Preparation……………………………………………………………………..4

III.References…………………………………………………………………………….18

IV. Supplementary tables……………………………………………………………………...19

V. *In Vitro* Experiments………………………………………………………………….25

I. General Information

H and C NMR spectra were recorded on Varian VNMRS 400 MHz, Varian Inova 500 MHz, and Bruker Avance III 600 MHz spectrometers at 25 ºC. All chemical shifts were reported in parts per million (ppm) and were internally referenced to residual protio solvents unless otherwise noted. Spectral data were reported as follows: chemical shift (multiplicity [singlet (s), doublet (d), triplet (t), quartet (q), pentet (p), and multiplet (m)], coupling constants [Hz], integration). Carbon spectra were recorded with complete proton decoupling. Conventional mass spectra were obtained using Advion Expression CMS (APCI and ASAP). All chemicals used in the synthesis of substrates were purchased from Sigma Aldrich, TCI, Frontier Scientific, Acros Organics, Strem, Oakwood, Matrix Scientific, or Fisher Scientific and were used as received without further purification, unless specifically noted. All normal phase flash column chromotagraphy (FCC) was performed using Grade 60 Silca Gel (230-400 mesh) purchased from Fisher Scientific or performed on a Biotage Isolera One with a Biotage SNAP cartridge (KP-SIL- 10-100g). Preparative Thin Layer Chromatography (TLC) plates contained grade 60 silica-gel coated with fluorescent indicator F_{254}. Atropisomerically stable compounds were isolated via chiral HPLC using an Agilent 1100 series HPLC. CHIRALPAK IA column were purchased from Diacel Technologies Corporation. Monowave 400 by Anton Paar was used for all microwave reactions in 10G or 30G reactor vessels.

Safety: no unexpected or unusually high safety hazards were encountered during the substrate preparation.

I. Substrate Preparation

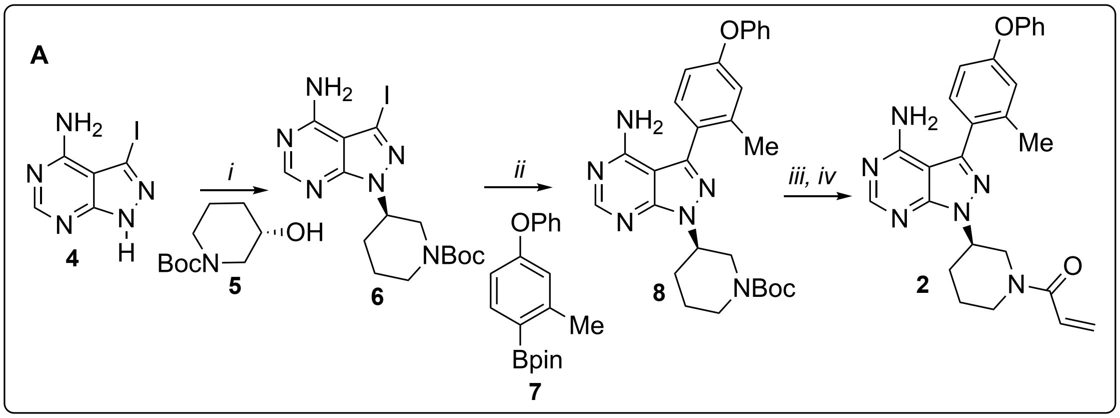

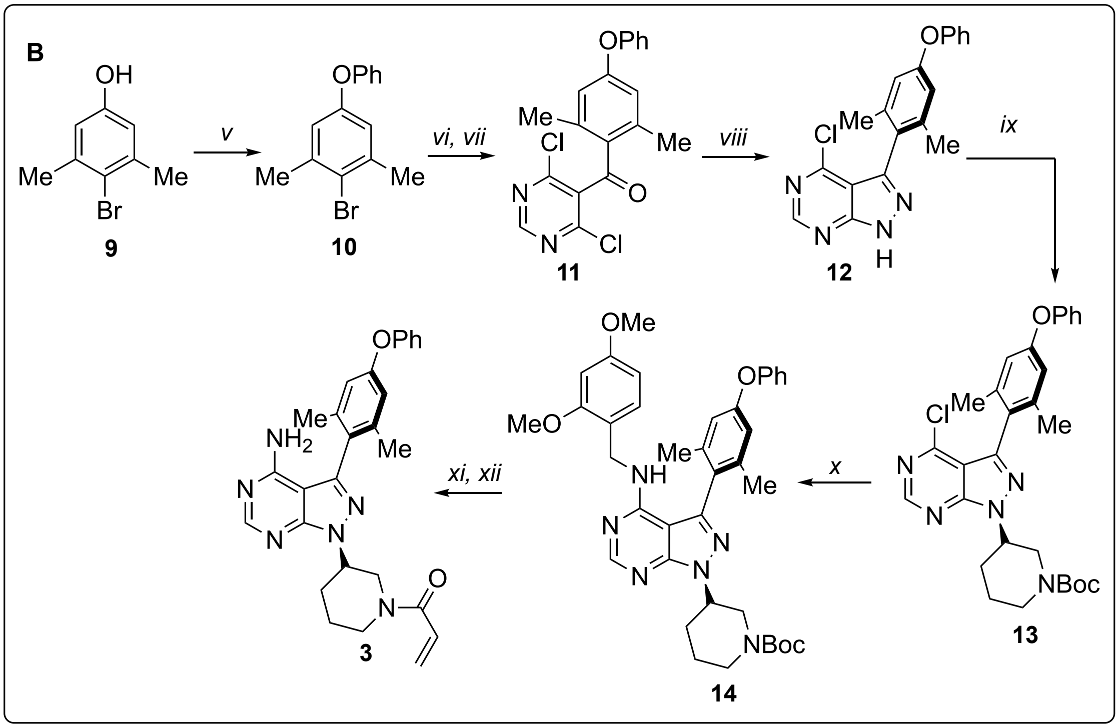

Substrate Preparation for 2:

Step i:

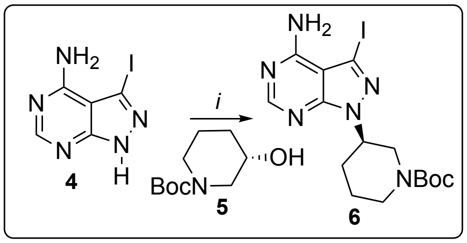

tert-butyl (R)-3-(4-amino-3-iodo-1H-pyrazolo[3,4-d]pyrimidin-1-yl)piperidine-1-carboxylate:

3-iodo-1H-pyrazolo[3,4-d]pyrimidin-4-amine, **4**, (2 g, 7.66 mmol, 1 eq.), tert-butyl (S)-3-hydroxypiperidine-1-carboxylate (4.62 g, 23.0 mmol, 3 eq.), **5**, and triphenylphosphine (4.02 g, 15.32 mmol, 2 eq.) were added to anhydrous THF (77 mL, 0.1 M) in a dried round bottom flask. After cooling the mixture to 0º C, DIAD (3 mL, 15.32 mmol, 2.0 eq.) was added and stirred for 10 minutes. The reaction mixture was then warmed to room temperature and stirred overnight. The reaction mixture was partitioned in ethyl acetate and brine. The organic layer was dried over sodium sulfate and concentrated. The crude extract was purified via FCC (96:4, DCM/MeOH) to yield 3.2 g of tert-butyl (R)-3-(4-amino-3-iodo-1H-pyrazolo[3,4-d]pyrimidin-1-yl)piperidine-1-carboxylate, **6** as a yellow solid (94% yield). The ^{1}H matched those reported in the literature.[6]

Steps towards generation of intermediate 7:

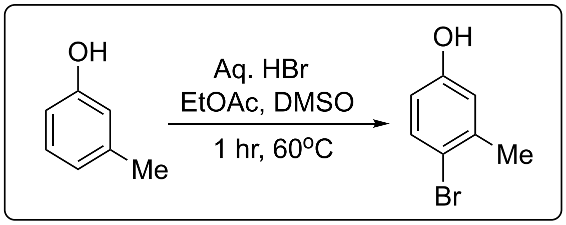

4-bromo-3-methylphenol:

M-cresol (4 g, 36.99 mmol, 1 eq.) and dimethyl sulfoxide (3.94 mL, 55.48 mmol, 1.5 eq.) was dissolved in ethyl acetate (148 mL, 0.25 M) at room temperature. Aqueous HBr 48% w/w (7.53 mL, 55.48 mmol, 1.5 eq.) was then added dropwise and the reaction was stirred for 1 hr at 60º C. Saturated aqueous sodium bicarbonate was added to quench the reaction. The reaction mixture was partitioned in ethyl acetate and brine. The organic layer was dried over sodium sulfate and concentrated. The crude extract was purified via FCC with a gradient of hexanes/ethyl acetate (100:0 to 70:30) to yield 5.13 g of pure 4-bromo-3-methylphenol as a yellow oil (74.2% yield). The ^{1}H NMR matched those reported in the literature.[2]

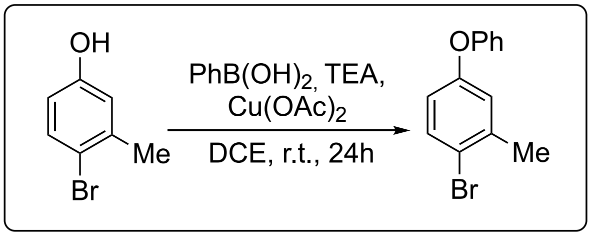

1-bromo-2-methyl-4-phenoxybenzene:
4Å powder molecular sieves (6.42 g, 200mg/mmol of $PhB(OH)_2$), were added to a round bottom flask and flame dried under vacuum. 107 mL (0.1M) of 1,2-dichloroethane was then added to the dried round bottom then opened to air. The functionalized phenol, 4-bromo-3-methylphenol (2 g, 10.69 mmol, 1 eq.), $PhB(OH)_2$ (3.91 g, 32.08 mmol, 3.0 eq.), triethylamine (7.45 mL, 53.45 mmol, 5.0 eq.), and copper (II) acetate anhydrous (3.88 g, 21.38 mmol, 2 eq.) were added and let stir overnight at room temperature. The crude mixture was filtered through celite to remove sieves and washed with dichloromethane. The reaction mixture was partitioned between dichloromethane and brine and washed with aqueous 30% ammonium hydroxide. The organic layer was dried over sodium sulfate and concentrated. The crude extract was purified by FCC with a gradient of hexanes/ethyl acetate (100:0 to 75:25), yielding 2.59 g of 1-bromo-2-methyl-4-phenoxybenzene as a white solid (92.5% yield). The ^1H NMR matched those reported in the literature.[3]

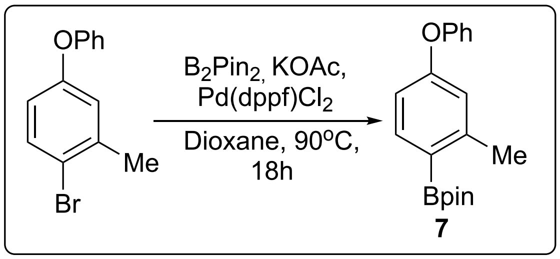

7

4,4,5,5-tetramethyl-2-(2-methyl-4-phenoxyphenyl)-1,3,2-dioxaborolane:
1-bromo-2-methyl-4-phenoxybenzene (2.59 g, 9.84 mmol, 1.0 eq.), bis(pinacalato)diboron (4.99 g, 19.69 mmol, 2.0 eq.), $Pd(dppf)Cl_2$ (402 mg, 0.492 mmol, 0.05 eq.), and potassium acetate (2.89 g, 29.52 mmol, 3.0 eq.) were added to a round bottom equipped with a stir bar. After purging reaction vessel with argon, degassed dioxane (33 mL, 0.3 M), was added and the reaction was refluxed at 90º C for 18 hr. The crude reaction mixture was filtered through celite and washed with ethyl acetate. The reaction mixture was partitioned between ethyl acetate and brine. The organic layer was dried over sodium sulfate and concentrated. The crude extract was purified by FCC with a gradient of hexanes/ethyl acetate (100:0 to 85:15), yielding 2.0 g of 4,4,5,5-tetramethyl-2-(2-methyl-4-phenoxyphenyl)-1,3,2-dioxaborolane as a clear oil (66% yield). The ^1H NMR matched those reported in the literature.[5]

Steps ii, iii, and iv:

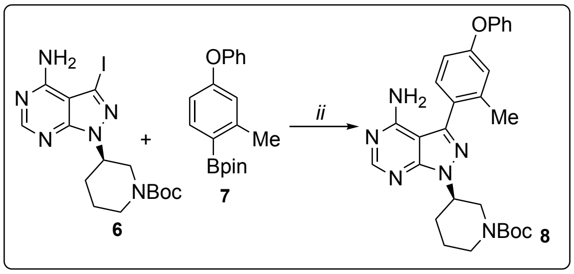

***tert-butyl* (R)-3-(4-amino-3-(2-methyl-4-phenoxyphenyl)-1H-pyrazolo[3,4-d]pyrimidin-1-yl)piperidine-1-carboxylate**:
6 (272 mg, 0.61 mmol, 1 eq.) was combined with boronic ester **7** (200 mg, 0.67 mmol, 1.1 eq.), $Pd(PPh_3)_4$ (70.5 mg, 0.061 mmol, 0.1 eq.), and K_2CO_3 (194 mg,1.4 mmol, 2.3 eq.). The reaction vessel was then purged with argon and a 3:1 mixture of dioxane/DI water (2.4 mL, 0.25 M), was added. The reaction was then stirred at 100º C for 18 hr. The crude reaction mixture was filtered through celite and washed with ethyl acetate. The crude mixture was then partitioned between ethyl acetate and brine. The organic later was dried over sodium sulfate and concentrated to yield crude **8**. The crude extract was then carried forward to the next reaction with no further purification due to the similarity in polarity of product and dehalogenated starting material.

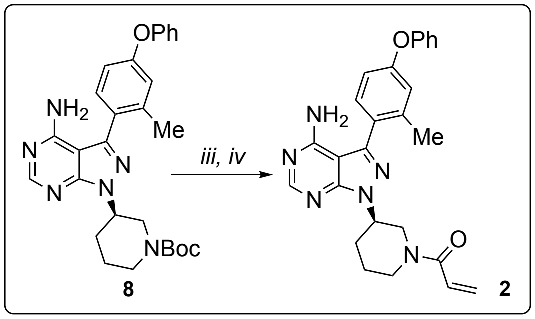

(R)-1-(3-(4-amino-3-(2-methyl-4-phenoxyphenyl)-1H-pyrazolo[3,4-d]pyrimidin-1-yl)piperidin-1-yl)prop-2-en-1-one:
8 (300mg, 0.6 mmol, 1.0 eq.) and TFA (500 uL, 1.2 M) were dissolved in 1,2-dichloroethane (2 mL, 0.12 M) and heated at 60º C for 1.5 hr. The crude mixture was partitioned in ethyl acetate and sodium bicarbonate. The organic layer was dried over sodium sulfate and concentrated. The crude extract (250 mg, 0.62 mmol, 1.0 eq.) was then dissolved in THF (12.4 mL, 0.05 M) followed by addition of DIPEA (650 uL, 3.74 mmol, 6 eq.). After the reaction mixture was cooled to 0 ºC, acryloyl chloride (66 uL, 0.81 mmol, 1.3 eq.) was added dropwise and stirred for 2 hours at 0º C. The crude mixture was partitioned in ethyl acetate and brine. The organic layer was dried over sodium sulfate and concentrated. The crude extract purified by FCC with a hexanes/ethyl acetate gradient (60:40 to 0:100) followed by DCM/MeOH (100:0 to 90:10) gradient, to yield 50 mg of **2** (18% yield).

MS (APCI) Calculated: $C_{26}H_{26}N_6O_2$ $[M+H]^+$ 455.2195 **Found:** 455.2190 m/z

^{1}H NMR: (400 MHz, Chloroform-*d*) δ 8.34 (s, 1H), 7.41 – 7.32 (m, 3H), 7.26 ($CDCl_3$, s) 7.16 (t, *J* = 7.6 Hz, 1H), 7.07 (d, *J* = 8.0 Hz, 2H), 6.99 (s, 1H), 6.94 (dd, *J* = 8.5, 2.5 Hz, 1H), 6.59 (dd, *J* =41.1, 12.3 Hz, 1H), 6.28 (d, *J* = 16.8 Hz, 1H), 6.20-5.16 (broad obscured singlet, 2H, NH_2), 5.62– 5.75 (m, 1H), 4.87 (d, J=8.0 Hz, 1H), 4.60 (d, J=12.8 Hz, 1H), 4.14 (dd, J=81.6, 56.0 Hz, 1H), 3.73 (t, J=11.4 Hz, 1H), 3.10–3.39 (m, 1H), 2.85 (t, J=11.7 Hz, 1H), 2.31–2.44 (m, 1H), 2.27 (s, 3H), 1.98 (d, *J* = 13.5 Hz, 1H), 1.71 (dd, J=38.5, 12.3 Hz, 1H).

^{13}C: (101 MHz, $CDCl_3$) δ 165.71, 158.43, 157.82, 156.34, 155.80, 153.65, 143.29, 139.72, 131.23, 129.90, 128.04, 127.60, 126.52, 126.35, 123.92, 120.70, 119.77, 119.51, 116.09, 99.61, 77.33, 77.22, 77.01, 76.70, 53.33, 46.19, 45.89, 29.66, 23.93, 20.12.

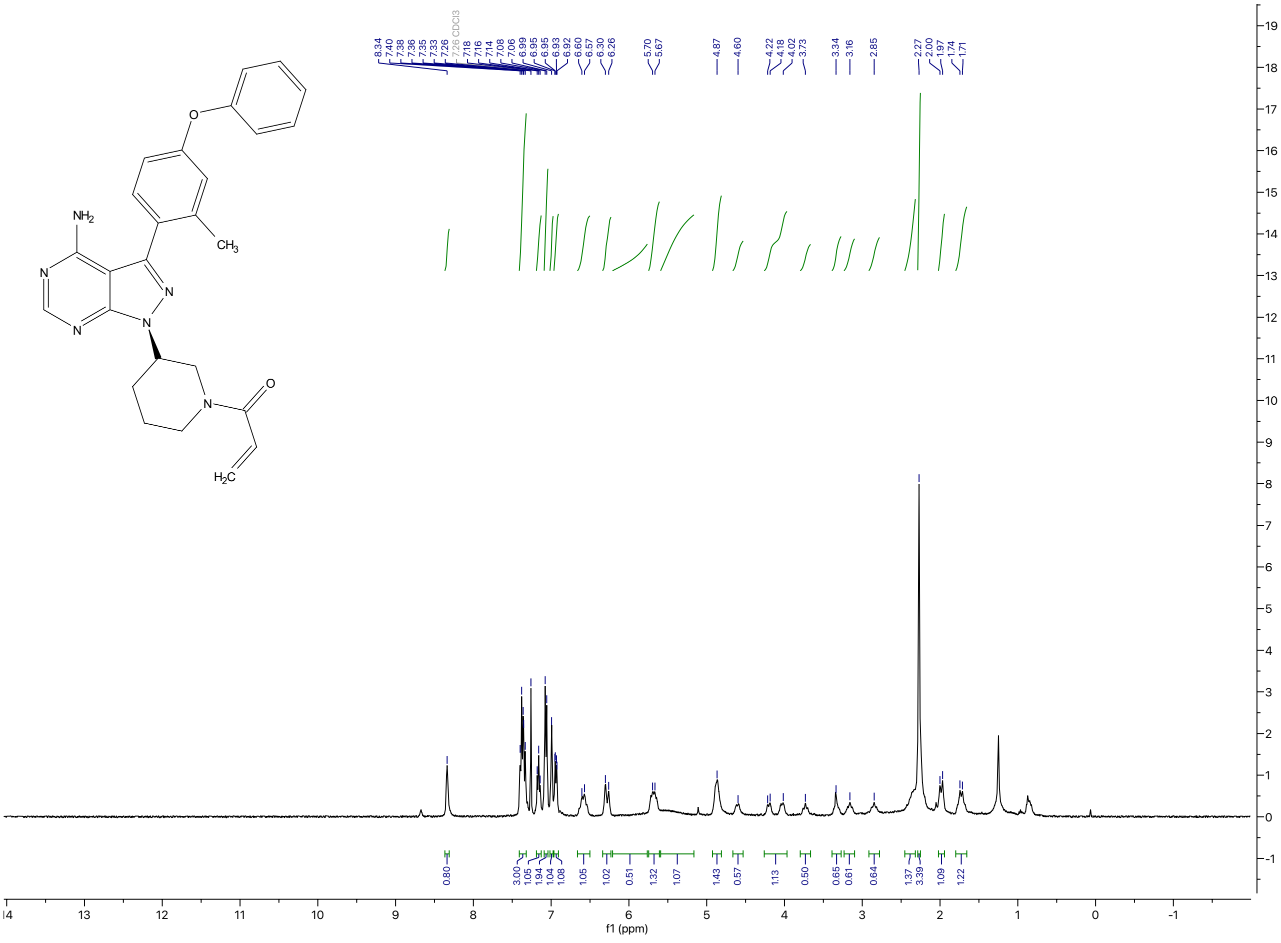

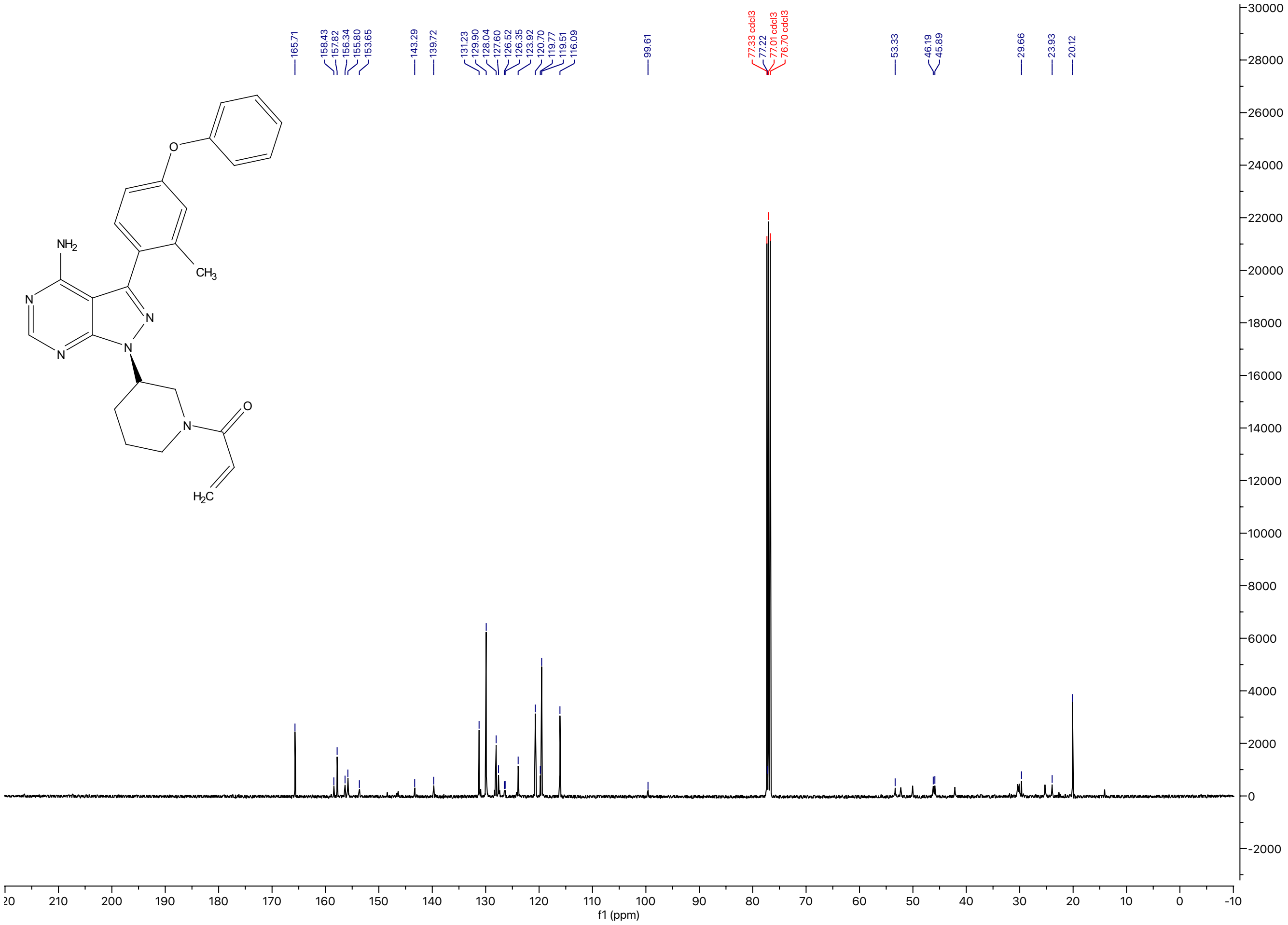

HPLC Trace: 2 was measured with HPLC analysis using Chiralpak IA Hexanes/EtOH (60:40), flow rate=1.5 mL/min, injection volume=20 ul.

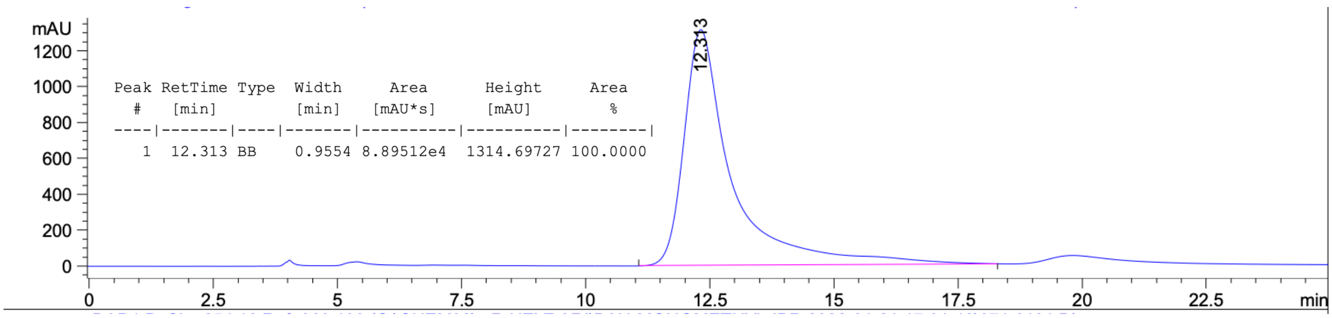

Substrate Preparation for 3:

Step v:

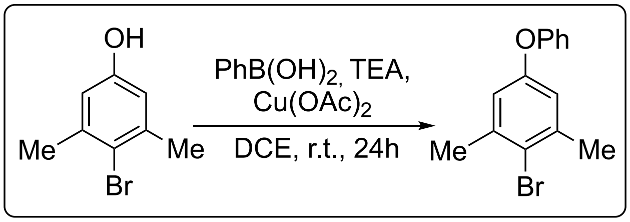

2-bromo-1,3-dimethyl-5-phenoxybenzene

4Å powder molecular sieves (15 g, 200mg/mmol of $PhB(OH)_2$), were added to a round bottom flask and flame dried under vacuum. 250 mL (0.1 M) of 1,2-dichloroethane was then added to the dried round bottom then opened to air. The functionalized phenol, 4-bromo-3,5-dimethylphenol (5 g, 24.88 mmol, 1.0 eq.), $PhB(OH)_2$ (9.1 g, 74.63 mmol, 3.0 eq.), triethylamine (17.3 mL, 124.4 mmol, 5.0 eq.), and copper (II) acetate anhydrous (9.04 g, 49.76 mmol, 2 eq.) were added and let stir overnight at room temperature. The crude mixture was filtered through celite to remove sieves and washed with dichloromethane. The reaction mixture was partitioned between dichloromethane and brine and washed with aqueous 30% ammonium hydroxide. The organic layer was dried over sodium sulfate and concentrated. The crude extract was purified by FCC with a gradient of hexanes/ethyl acetate (100:0 to 75:25), yielding 3.3 g of 2-bromo-1,3-dimethyl-5-phenoxybenzene (48% yield). The ^1H NMR spectra matched those reported in the literature.[4]

Steps vi and vii:

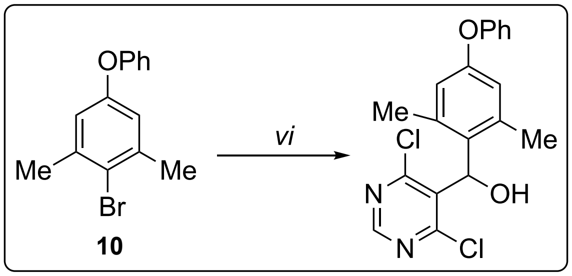

4,6-dichloropyrimidin-5-yl)(2,6-dimethyl-4-phenoxyphenyl)methanol:

2-bromo-1,3-dimethyl-5-phenoxybenzene (1.58 g, 5.77 mmol, 1.5 eq.) was added to activated magnesium turnings (0.415 g, 17.31 mmol, 4.5 eq.) in dry diethyl ether (23 mL, 0.25 M) in a flame-dried round bottom flask under argon. The reaction was stirred at room temperature overnight. In a separate flame-dried round bottom flask 2-(4,6-dichloropyrimidin-5-yl)acetaldehyde (0.677 g, 3.85 mmol, 1.0 eq.) was dissolved into 3.9 mL of diethyl ether (1.0 M) under an argon atmosphere. The reaction flask was cooled down to -78º C. The magnesium bromide aryl Grignard (23 mL) was added dropwise and let stir for 0.5 hr at -78º C. The reaction was let to warm to room temperature and stirred for an additional 3 hours, then quenched with water. The reaction mixture was

partitioned in ethyl acetate and brine. The organic layer was dried over sodium sulfate and concentrated. The crude extract was purified via FCC (80:20, hexanes/ethyl acetate gradient) to yield 0.760 g of pure (4,6-dichloropyrimidin-5-yl)(2,6-dimethyl-4-phenoxyphenyl)methanol as a white solid (53% yield).
^{1}**H NMR**: (400 MHz, Chloroform-*d*) δ 8.70 (s, 1H), 7.40 – 7.29 (m, 2H), 7.26 ($CDCl_3$, s) 7.12 (t, J = 7.4 Hz, 1H), 7.01 (d, J = 7.7 Hz, 2H), 6.66 (s, 2H), 6.47 (d, J = 7.4 Hz, 1H), 2.57 (d, J = 7.4 Hz, 1H), 2.27 (s, 6H).
MS (APCI) Calculated: $C_{19}H_{17}Cl_2N_2O_2$ $[M+H]^+$ 375.1 **Found:** 375.0 m/z

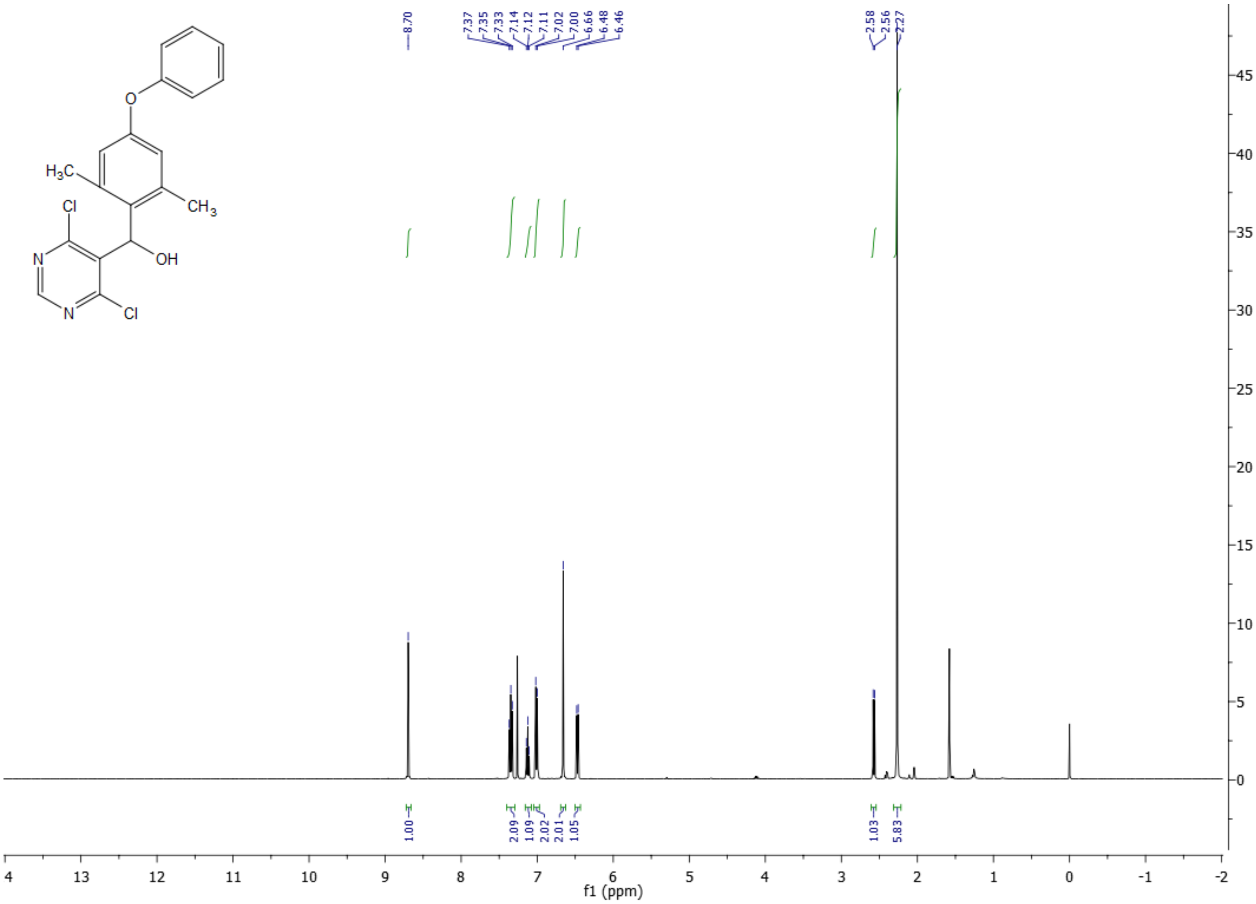

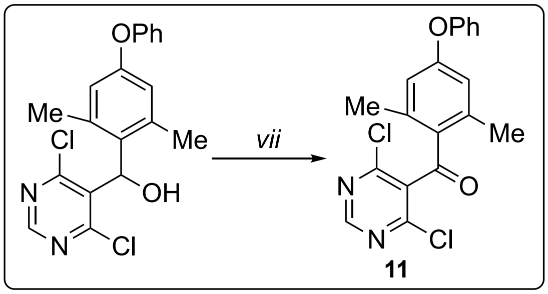

11

(4,6-dichloropyrimidin-5-yl)(2,6-dimethyl-4-phenoxyphenyl)methanone:
To (4,6-dichloropyrimidin-5-yl)(2,6-dimethyl-4-phenoxyphenyl)methanol (0.760 g, 2.025 mmol, 1.0 eq.), Dess-Martin periodinane (1.72 g, 4.05 mmol, 2.0 eq.) was added and dissolved into 4 ml of DCM (0.5 M). The reaction was left to stir overnight. The reaction mixture was partitioned in DCM, sodium thiosulfate (aq.) and sodium bicarbonate (aq.) The organic layer was dried over sodium sulfate and concentrated. The crude extract was purified via FCC (80:20, hexanes/ethyl acetate) to yield 0.590 g of pure (4,6-dichloropyrimidin-5-yl)(2,6-dimethyl-4-phenoxyphenyl)methanone as a white solid (78% yield).
^{1}**H NMR**: (400 MHz, Chloroform-*d*) δ 8.82 (s, 1H), 7.40 (t, *J* = 8.0 Hz, 2H), 7.26 ($CDCl_3$, s), 7.20 (t, *J* = 7.4 Hz, 1H), 7.07 (d, *J* = 7.6 Hz, 2H), 6.66 (s, 2H), 2.24 (s, 6H).
MS (APCI) Calculated: $C_{19}H_{15}Cl_2N_2O_2$ $[M+H]^+$ 373.1 **Found:** 373.1 m/z

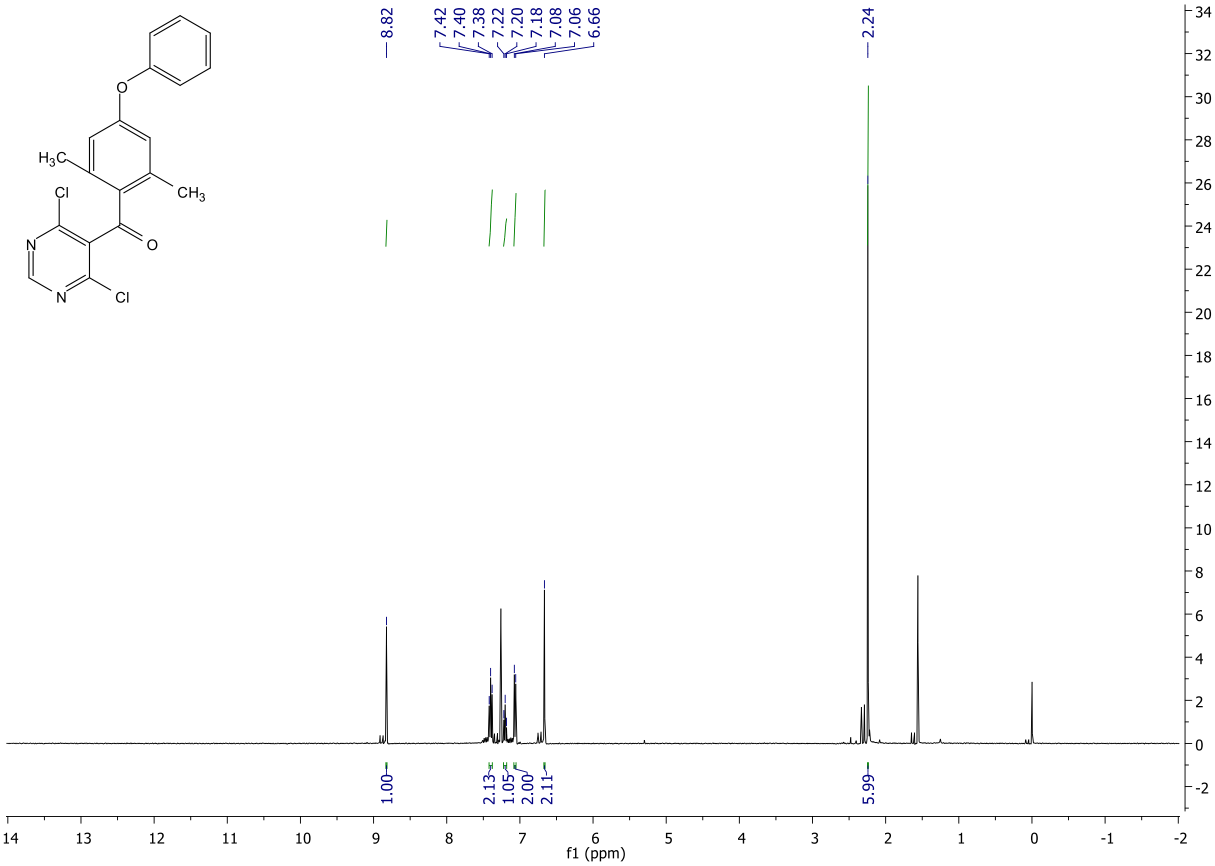

Step viii:

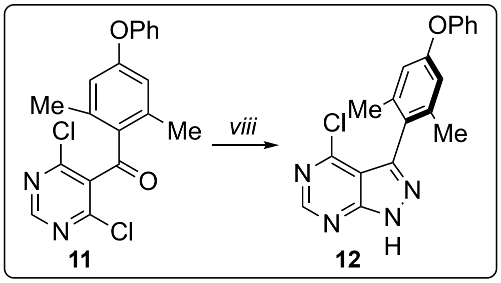

4-chloro-3-(2,6-dimethyl-4-phenoxyphenyl)-1H-pyrazolo[3,4-d]pyrimidine:
To (4,6-dichloropyrimidin-5-yl)(2,6-dimethyl-4-phenoxyphenyl)methanone (0.590 g, 1.58 mmol, 1 eq.), triethylamine (0.218 mL, 1.58 mmol, 1 eq.) and hydrazine monohydrate (0.083 mL, 1.74 mmol, 1.1 eq.) was dissolved into THF (8 mL, 0.2 M) in a round bottom flask. After stirring for one hour at room temperature the reaction was heated to 65 ºC and stirred overnight. The reaction mixture was partitioned in ethyl acetate and brine. The organic layer was dried over sodium sulfate and concentrated. The crude extract was purified via FCC (70:30, hexanes/ethyl acetate) to yield 0.298 g of 4-chloro-3-(2,6-dimethyl-4-phenoxyphenyl)-1H-pyrazolo[3,4-d]pyrimidine as a yellow solid (54% yield).
^{1}**H NMR**: (400 MHz, Chloroform-*d*) δ 11.72 (s, 1H), 8.89 (s, 1H), 7.43 – 7.33 (m, 2H), 7.26 ($CDCl_3$, s), 7.15 (t, J = 7.4 Hz, 1H), 7.11 – 7.07 (m, 2H), 6.81 (s, 2H), 2.03 (s, 6H).
MS (APCI) Calculated: $C_{19}H_{16}ClN_4O$ $[M+H]^+$ 351.1 **Found:** 351.1 m/z

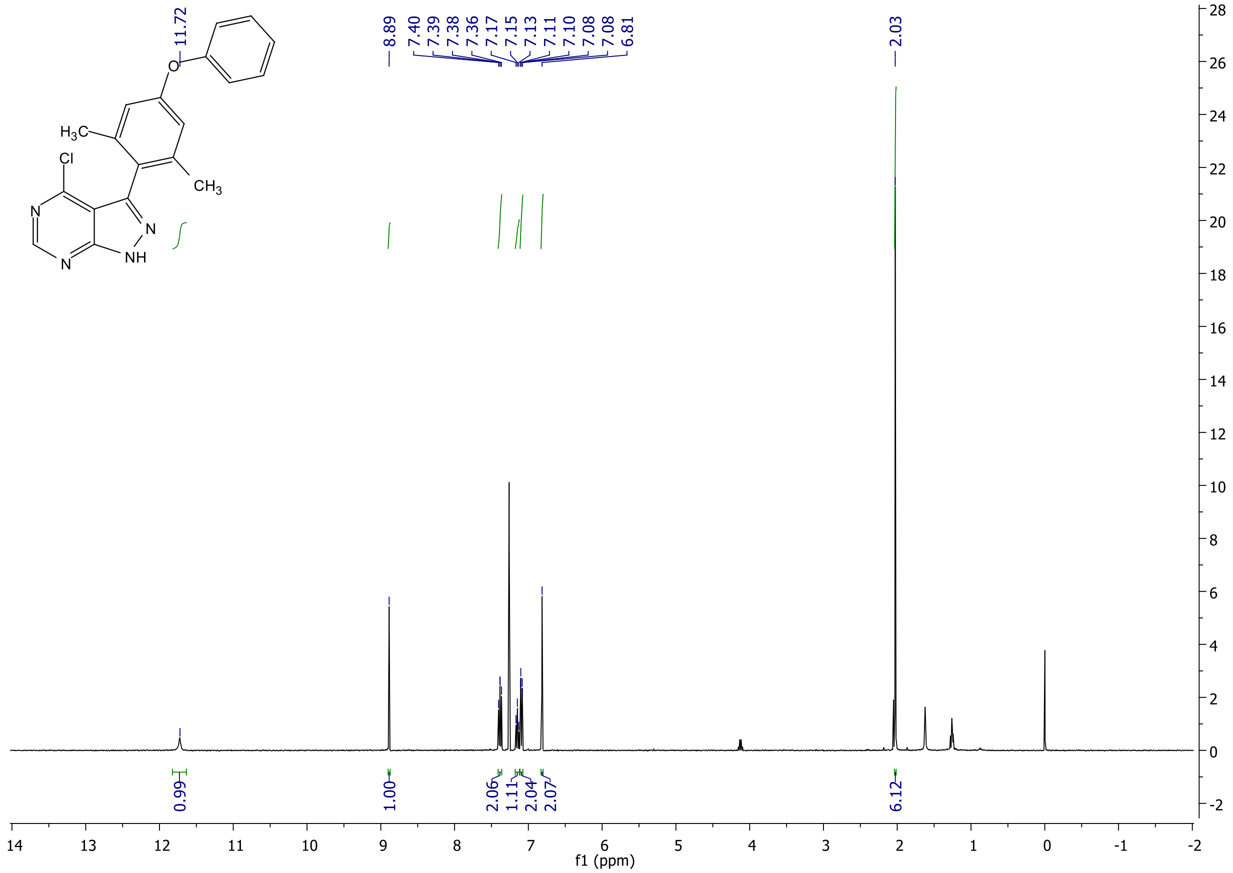

Step ix:

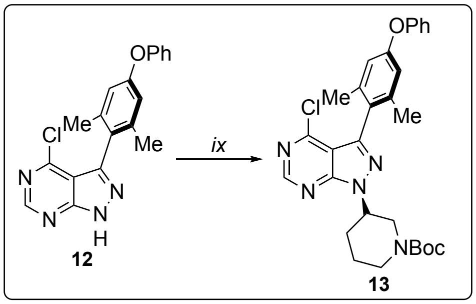

tert-butyl (R)-3-(4-chloro-3-(2,6-dimethyl-4-phenoxyphenyl)-1H-pyrazolo[3,4-d]pyrimidin-1-yl)piperidine-1-carboxylate:

4-chloro-3-(2,6-dimethyl-4-phenoxyphenyl)-1H-pyrazolo[3,4-d]pyrimidine (0.178 g, 0.509 mmol, 1 eq.), tert-butyl (R)-3-hydroxypiperidine-1-carboxylate (0.307 g, 1.53 mmol, 3 eq.), and triphenylphosphine (0.267 g, 1.02 mmol, 2 eq.) were added to anhydrous THF (5 mL, 0.1 M) in a dried round bottom flask. After cooling the mixture to 0º C, DIAD (0.200 mL, 1.02 mmol, 2 eq.) was added and stirred for 10 minutes. The reaction mixture was then warmed to room temperature and stirred overnight. The reaction mixture was partitioned in ethyl acetate and brine. The organic layer was dried over sodium sulfate and concentrated. The crude extract was purified via FCC (96:4 DCM/MeOH) to yield 0.171 g of tert-butyl (R)-3-(4-chloro-3-(2,6-dimethyl-4-phenoxyphenyl)-1H-pyrazolo[3,4-d]pyrimidin-1-yl)piperidine-1-carboxylate as a yellow solid (63% yield).

^{1}H NMR: (400 MHz, Chloroform-*d*) δ 8.78 (s, 1H), 7.39 – 7.36 (m, 2H), 7.26 ($CDCl_3$, s), 7.14 (t, *J* = 7.4 Hz, 1H), 7.07 (d, *J* = 7.6 Hz, 2H), 6.80 (s, 2H), 4.96 (s, 1H), 4.35 (s, 1H), 4.12 (s, 1H), 3.41 (s, 1H), 2.85 (t, *J* = 10.9 Hz, 1H), 2.30 (d, *J* = 7.5 Hz, 1H), 2.25 – 2.19 (m, 1H), 2.00 (s, 6H), 1.91 (d, *J* = 16.4 Hz, 1H), 1.77 – 1.70 (m, 1H), 1.47 (s, 9H).

MS (APCI) Calculated: $C_{29}H_{32}ClN_5O_3$ [M] 533.2 **Found:** 533.2 m/z

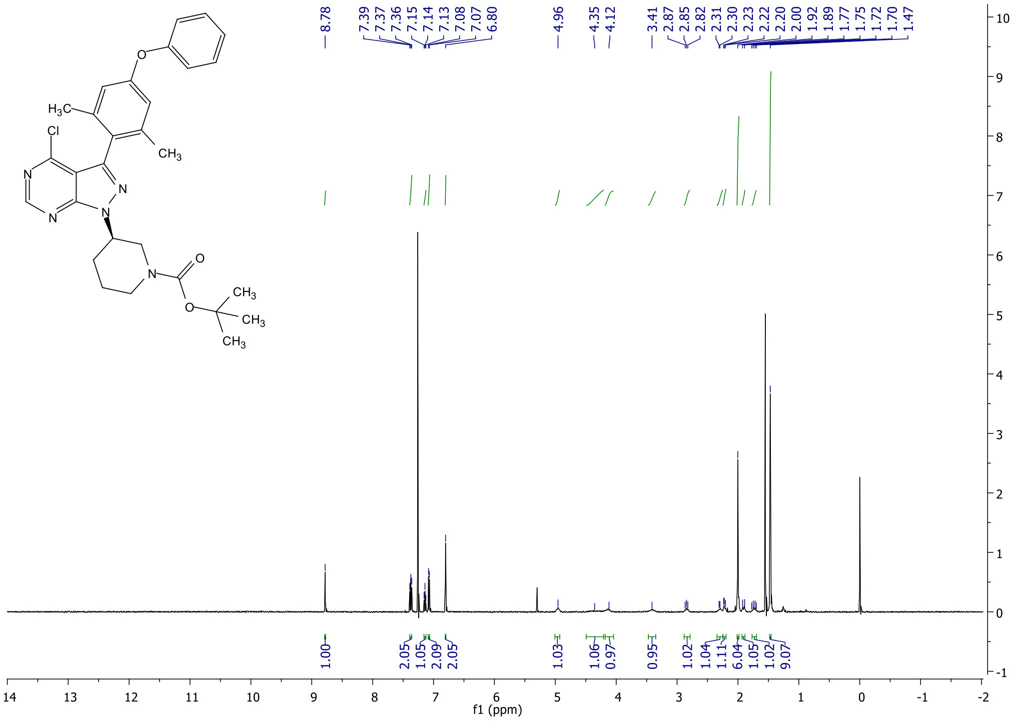

Step x:

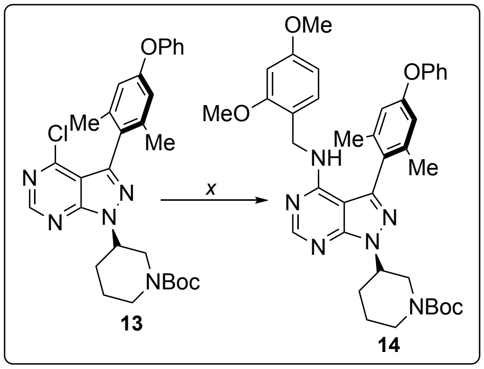

tert-butyl (R)-3-(4-((2,4-dimethoxybenzyl)amino)-3-(2,6-dimethyl-4-phenoxyphenyl)-1H-pyrazolo[3,4-d]pyrimidin-1-yl)piperidine-1-carboxylate:
13 (171 mg, 0.32 mmol, 1 eq.) was aminated with 2,4-dimethoxybenzylamine (100 uL, 0.67 mmol, 2.0 eq.) using Cs_2CO_3 (240 mg, 0.74 mmol, 2.3 eq.) in dioxanes (1.6 mL, 0.2 M), and refluxed at 80º C for 18 hr. The crude mixture was partitioned in ethyl acetate and brine. The organic layer was dried over sodium sulfate and concentrated. The crude extract was purified by FCC with a hexanes/ethyl acetate gradient (80:20 to 0:100), to yield 105 mg of **14** as a yellow powder (49% yield).
^{1}H NMR: (400 MHz, Chloroform-*d*) δ 8.44 (s, 1H), 7.39 – 7.35 (m, 2H), 7.26 ($CDCl_3$, s), 7.20 (s, 1H), 7.15 (t, J = 7.4 Hz, 1H), 7.01 (d, J = 7.7 Hz, 2H), 6.78 (s, 2H), 6.39 (dq, J = 4.9, 2.4 Hz, 2H), 5.44 (s, 1H), 4.81 (dt, J = 7.8, 6.1 Hz, 1H), 4.61 (s, 2H), 4.37 (s, 1H), 4.20 (s, 1H), 3.77 (s, 3H), 3.65 (s, 3H), 3.35 (s, 1H), 2.77 (t, J = 10.0 Hz, 1H), 2.24 – 2.12 (m, 2H), 1.97 (s, 6H), 1.88 – 1.81 (m, 1H), 1.73 – 1.65 (m, 1H), 1.45 (s, 9H).
MS (APCI) Calculated: $C_{38}H_{45}N_6O_5$ $[M+H]^+$ 665.3 **Found:** 665.4 m/z

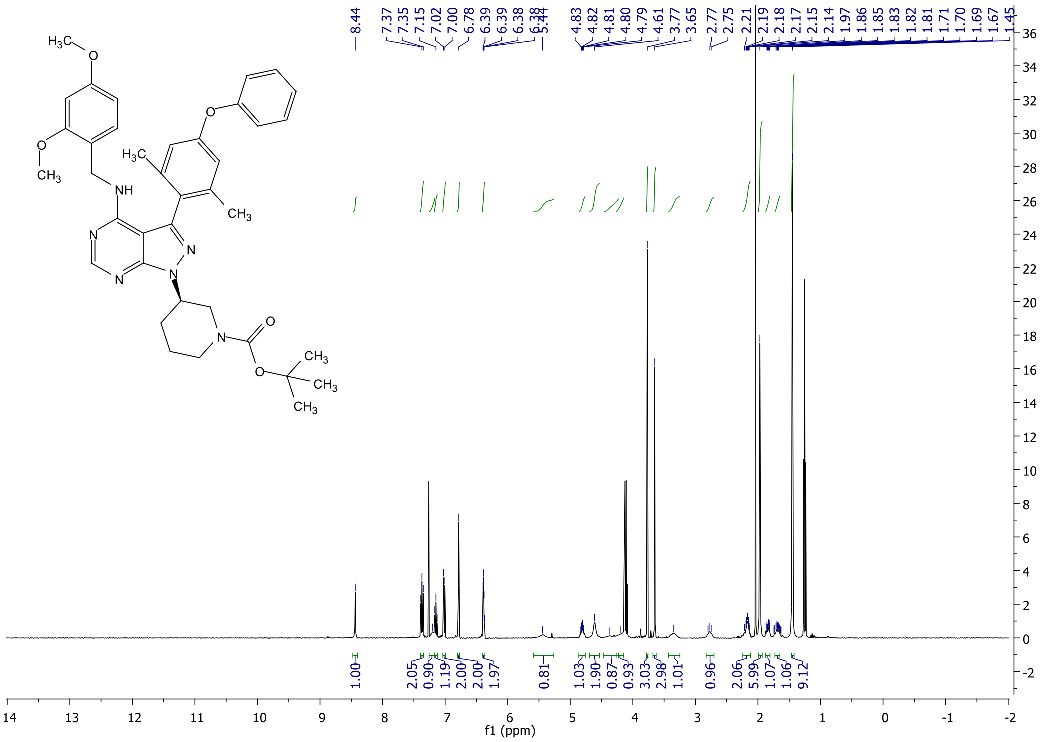

Steps xi and xii:

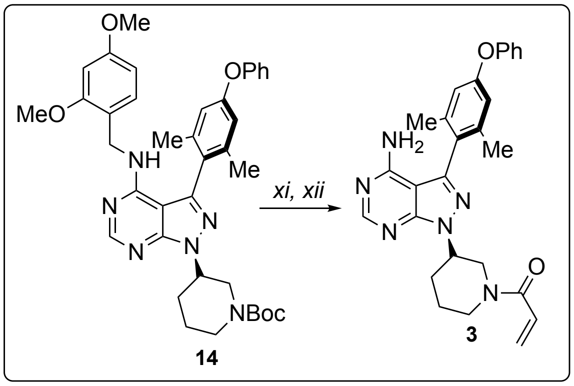

(R)-1-(3-(4-amino-3-(2-methyl-4-phenoxyphenyl)-1H-pyrazolo[3,4-d]pyrimidin-1-yl)piperidin-1-yl)prop-2-en-1-one:
14 (105 mg, 0.158 mmol, 1 eq), and TFA (1.05 mL, 0.15 M) were dissolved in DCE (1.05 mL, 0.15 M) and heated at 60º C for 1.5 hr. The crude mixture was partitioned in ethyl acetate and sodium bicarbonate. The organic layer was dried over sodium sulfate and concentrated. The crude extract was then dissolved in THF (2 mL, 0.05 M) followed by addition of DIPEA (200 uL, 1.2 mmol, 6 eq.). After the reaction mixture was cooled to 0 ºC, acryloyl chloride (19 uL, 0.21 mmol, 1.1 eq.) was added dropwise and stirred for 2 hours at 0º C. The crude mixture was partitioned in ethyl acetate and brine. The organic layer was dried over sodium sulfate and concentrated. The crude extract purified by FCC with a hexanes/ethyl acetate gradient (60:40 to 0:100) followed by DCM/MeOH (100:0 to 90:10) gradient, to yield 32mg of **3** as a white powder (43% yield).

^{1}H NMR: ^{1}H NMR (400 MHz, $CDCl_3$) δ 8.38 (s, 1H), 7.46 – 7.39 (m, 2H), 7.26 ($CDCl_3$, s), 7.20 (t, *J* = 7.4 Hz, 1H), 7.11 (d, *J* = 7.6 Hz, 2H), 6.87 (s, 2H), 6.70 – 6.60 (m, 1H), 6.34 (dd, *J* = 16.8, 2.0 Hz, 1H), 6.23-4.94 (broad obscured singlet, 2H, NH_2), 5.74 (t, *J* = 12.5 Hz, 1H), 4.93 (d, J=10.3 Hz, 1H), 4.66 (d, *J* = 11.6 Hz, 1H), 4.17 (dd, *J* = 64.7, 13.8 Hz, 1H), 3.89 – 3.75 (m, 1H), 3.30 (dt, *J* = 76.7, 11.0 Hz, 1H), 2.89 (t, J=12.6 Hz, 1H), 2.35–2.25 (m, 1H), 2.11 (s, 6H), 2.03 (dt, *J* = 13.1, 3.3 Hz, 1H), 1.85 – 1.71 (m, 1H).

^{13}C NMR: (101 MHz, Chloroform-*d*) δ 166.12, 163.02, 162.64, 159.27, 155.79, 153.50, 151.13, 146.10, 145.57, 139.75, 139.44, 130.01, 128.99, 127.02, 124.29, 123.00, 119.94, 117.69, 114.24, 98.39, 53.25, 46.14, 45.92, 30.18, 24.95, 20.24, 20.21.

MS (APCI) Calculated: $C_{27}H_{29}N_6O_2$ $[M+H]^+$ 469.2352 **Found:** 469.2344 m/z

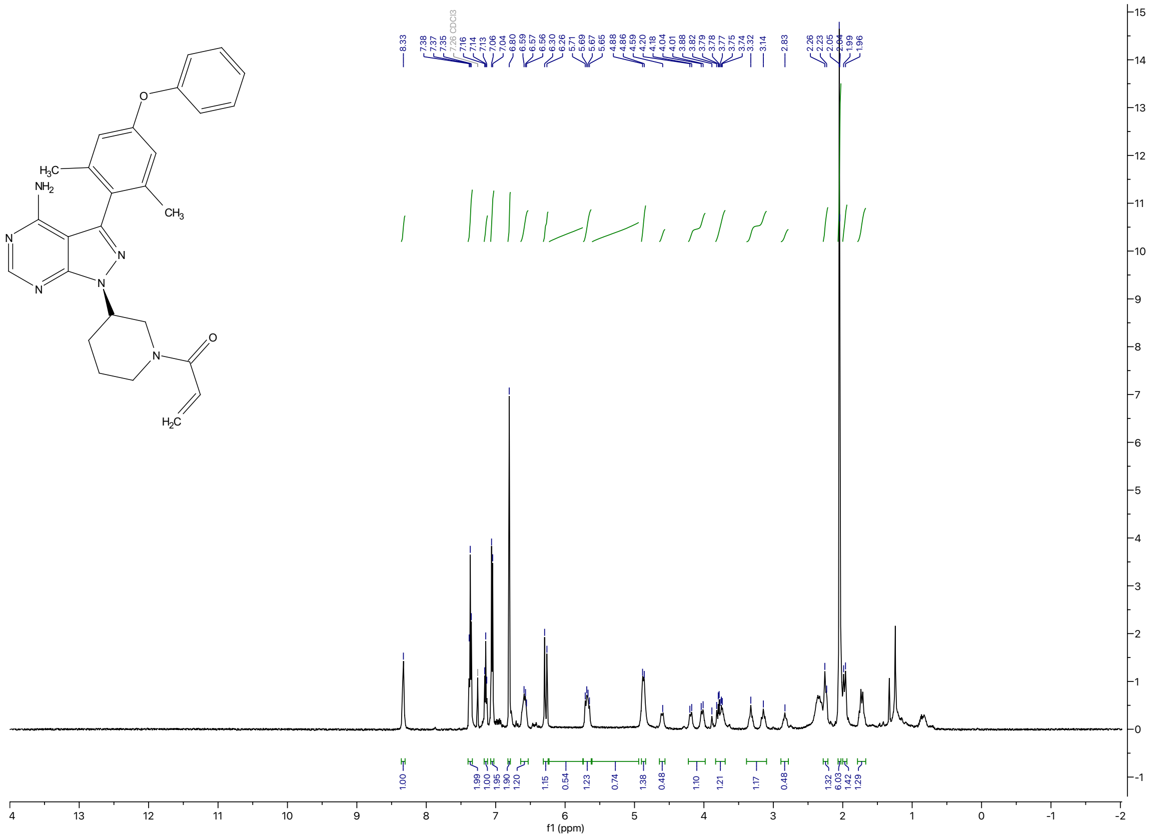

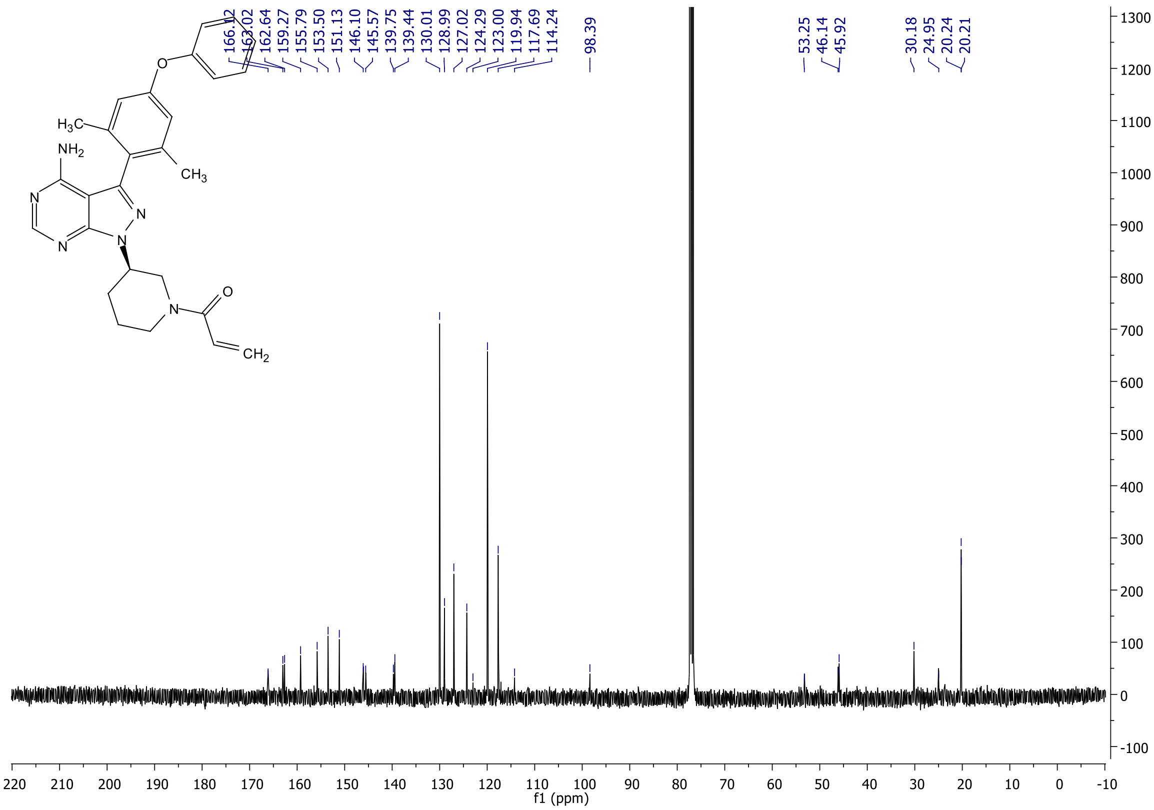

HPLC trace: **3** was measured with HPLC analysis using Chiralpak IA Hexanes/EtOH (50:50), flow rate=1.5 mL/min, injection volume=20 ul

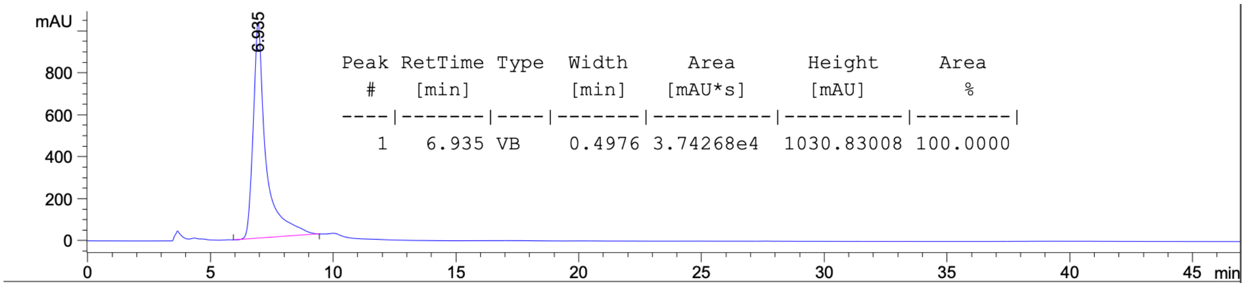

References:

(1) Frisch, M. J.; Trucks, G. W.; Schlegel, H. B.; Scuseria, G. E.; Robb, M. A.; Cheeseman, J. R.; Scalmani, G.; Barone, V.; Mennucci, B.; Petersson, G. A.; Nakatsuji, H.; Caricato, M.; Li, X.; Hratchian, H. P.; Izmaylov, A. F.; Bloino, J.; Zheng, G.; Sonnenb, D. J. No Title. *Gaussian 09, Revis. E.01* Gaussian Inc.: Wallingford, CT, 2009.

(2) Kavala, V.; Naik, S.; Patel, B. K. A New Recyclable Ditribromide Reagent for Efficient Bromination under Solvent Free Condition. *J. Org. Chem.* **2005**, *70*, 4267–4271.

(3) Bourbeau, M. P.; Siegmund, A.; Allen, J. G.; Shu, H.; Fotsch, C.; Bartberger, M. D.; Kim, K.; Komorowski, R.; Graham, M.; Busby, J.; et al. Piperazine Oxadiazole Inhibitors of Acetyl-CoA Carboxylase. **2013**.

(4) Lee, M.; Hesek, D.; Shi, Q.; Noll, B. C.; Fisher, J. F. Conformational Analyses of Thiirane-Based Gelatinase Inhibitors. **2008**, *18*, 3064–3067.

(5) Hirst, G. C.; Calderwood, D.; Munschaer, R.; Arnold, L. D.; Johnston, D. N.; Rafferty, P. Pyrrolopyrimidines as Tyrosine Kinase Inhibitors, 2001.

(6) Zapf, C. W.; Gerstenberger, B. S.; Xing, L.; Limburg, D. C.; Anderson, D. R.; Caspers, N.; Han, S.; Aulabaugh, A.; Kurumbail, R.; Shakya, S.; et al. Covalent Inhibitors of Interleukin - 2 Inducible T Cell Kinase (Itk) with Nanomolar Potency in a Whole-Blood Assay. **2012**.

I. Supplementary Tables

Supplementary Table 1: Low energy conformational energy profiles of **2**, **3**, and Ibrutinib. All structures were optimized in the gas phase using density functional theory (RB3LYP) with the 6-31G(d) basis set as implemented in the Gaussian 09[1] suite of programs. The indicated dihedral angle was frozen every 5 degrees while the rest of the structure was minimized and energies were recorded. All energies are reported in kcal/mol with respect to the global minimum of each inhibitor. The low energy conformational window (1.36 kcal/mol or lower) is highlighted in green.

	3	2	Ibrutinib
Dihedral Angle	Energy (kcal/mol)	Energy (kcal/mol)	Energy (kcal/mol)
-180	21.05	16.06	3.34
-175	18.13	13.56	2.53
-170	15.41	11.26	1.81
-165	12.92	9.19	1.19
-160	10.69	7.37	0.69
-155	8.73	5.79	0.32
-150	7.02	4.46	0.09
-145	5.40	3.31	0.00
-140	4.02	2.26	0.03
-135	2.87	1.43	0.18
-130	1.94	0.81	0.42
-125	1.21	0.37	0.75
-120	0.67	0.11	1.13
-115	0.30	0.00	1.55
-110	0.09	0.02	1.92
-105	0.00	0.13	2.27
-100	0.01	0.31	2.57
-95	0.07	0.50	2.78
-90	0.15	0.67	2.89
-85	0.20	0.75	2.78
-80	0.23	0.76	2.57
-75	0.29	0.71	2.27
-70	0.32	0.65	1.92
-65	0.41	0.60	1.55
-60	0.58	0.59	1.13
-55	1.03	0.65	0.75
-50	1.64	0.79	0.42
-45	2.44	0.93	0.18
-40	3.42	1.17	0.03
-35	4.60	1.57	0.00
-30	5.98	2.17	0.09
-25	7.57	2.93	0.32
-20	9.36	3.84	0.69
-15	11.36	4.90	1.19
-10	13.54	6.09	1.81
-5	15.90	7.40	2.53
0	18.34	8.79	3.34

Supplementary Table 2: Protein Data Bank ligand conformational analysis. Relative angles were used for Fig. 2 in manuscript:

PDB ID	Protein	Chemical ID	Scaffold	Angle	Relative Angle	Aryl substitution	Position
5OP5	CHK1	3FE	PPY	29.16	-150.84	CN	*m*
6BFA	CDPK1	UW5	PP	-143.27	-143.27	napth	*m*
4MX9	CDPK1	2E8	PP	-139.39	-139.39	napth	*m*
4W8E	MST3	3JB	PPY	42.94	-137.06	CN	*m*
5UNP	CLK2	8FY	PPY	-136.4	-136.4	Isoquinoline	*m*
3BHJ	CBR1	AB3	PP	-133.06	-133.06	OH	*m*
5GTY	Egfr (T790M)	816	PP	-132.66	-132.66	Cl	*m*
1WMA	Cbr1	AB3	PP	-132.64	-132.64	OH	*m*
4JBV	CDPK1	V68	PP	-132.55	-132.55	napth	*m*
2YM5	Chk1	YM5	PPY	-132.5	-132.5	MeOH	*m*
4N4S	ERk2	2H1	PP	48.32	-131.68	Me	*m*
3BHM	CBR1	AB3	PP	-130.91	-130.91	OH	*m*
3T3V	CDPK1	BK4	PP	-130.37	-130.37	napth	*m*
4TZR	CDPK1	UW2	PP	-130.36	-130.36	Isoquinoline	*m*
3V51	CDPK1	I76	PP	-130.14	-130.14	napth	*m*
4WG3	CDPK1	UWA	PP	50.69	-129.31	napth	*m*
4U8Z	MST3	3FE	PPY	51.24	-128.76	CN	*m*
3SXF	CDPK1	BK5	PP	-128.31	-128.31	napth	*m*
3I7C	CDPK1	BK2	PP	-127.29	-127.29	napth	*m*
3EN6	Src	KS5	PP	-126.51	-126.51	Isoquinoline	*m*
4DGG	Src	I76	PP	-125.76	-125.76	napth	*m*
3V5P	CDPK1	C88	PP	-125.71	-125.71	Me	*m*
3T3U	CDPK1	BK6	PP	-125.23	-125.23	napth	*m*
4MXA	CDPK1	BK7	PP	-124.95	-124.95	napth	*m*
2C0T	Hck	L3G	PP	-124.47	-124.47	OMe	*m*
3V5T	CDPK1	UW9	PP	-124.27	-124.27	napth	*m*
3VS6	Hck	VSH	PP	-123.28	-123.28	OMe	*m*
1YOL	Src	S03	PPY	-122.67	-122.67	OMe	*m*
3UQF	Src	BK5	PP	-121.97	-121.97	napth	*m*
3SX9	CDPK1	BK7	PP	-121.96	-121.96	napth	*m*
3EN7	Src	ABJ	PP	-121.4	-121.4	OH	*m*
5J9Y	Egfr (T790M)	6HL	PP	-120.67	-120.67	napth	*m*
5GNK	Egfr (T790M)	80U	PP	-119.31	-119.31	Cl	*m*
4WG4	CDPK1	UWB	PP	62.11	-117.89	napth	*m*
2C0O	Hck	L2G	PP	-117.02	-117.02	OMe	*m*
2C0I	Hck	L1G	PP	-116.57	-116.57	OMe	*m*
3EN5	Src	KS4	PP	-106.94	-106.94	OMe	*m*
4G31	PERK	0WH	PPY	-59.27	-59.27	indoline	*m*
4X7O	PERK	3Z6	PPY	-54.3	-54.3	indoline	*m*
2WXH	PI3Kd	ZZO	PP	-53.93	-53.93	F	*m*
2WXG	PI3Kd	ZZN	PP	-50.86	-50.86	F	*m*
5HHW	IGFR1	60O	PPY	-49.29	-49.29	O-alkyl	*m*
2V4L	PI3Kg	ABJ	PP	-47.76	-47.76	OH	*m*
3ENE	PI3Kg	NPZ	PP	-45.42	-45.42	napth	*m*
5HZN	IGFR1	66A	PPY	-41.17	-41.17	Obz	*m*
4HCT	ITK	18R	PP	-40.02	-40.02	amide	*m*
4HCV	ITK	13J	PP	-39.21	-39.21	amide	*m*
6G6W	PI3Kd	EO5	PPY	142.73	-37.27	sulfonamide	*m*
4HCU	ITK	13L	PP	-37.1	-37.1	amide	*m*
2WXJ	PI3Kd	RW3	PP	-33.81	-33.81	benathiozole	*m*
2WXK	PI3Kd	RW4	PP	-31.21	-31.21	benathiozole	*m*
4GKH	APH(3')-Ia	0J9	PP	34.17	-145.83	napth	*o*
6HJJ	Aurora	G7T	PP	46.34	-133.66	Me	*o*
4K11	Src (T338G)	0J9	PP	-127.97	-127.97	napth	*o*
3FI4	p38	FI4	PP	114.2	-65.8	Cl	*o*
4M7I	PERK	27D	PPY	-58.92	-58.92	F	*o*
4FEW	APH(3')-Ia	PP2	PP	-179.78	-179.78	Cl	*p*
4FEV	APH(3')-Ia	PP1	PP	-174.95	-174.95	Me	*p*
3VS2	Hck	VSB	PPY	-150.1	-150.1	OPh	*p*
3VS5	Hck	VSG	PPY	-143.66	-143.66	OPh	*p*
4CM1	PTR1	IQW	PPY	-137.94	-137.94	Me	*p*

2BRN	Chk1	DF1	PPY	-133.19	-133.19	H	*p*
3VRZ	Hck	VRZ	PPY	-131.65	-131.65	urea	*p*
3VS3	Hck	VSE	PPY	-131.14	-131.14	OPh	*p*
4CMB	PTR1	UEN	PPY	-130.85	-130.85	F	*p*
4EWH	Ack1	T77	PPY	-130.82	-130.82	H	*p*
5H0H	Hck	OOV	PPY	-130.55	-130.55	OPh	*p*
3VS0	Hck	VS0	PPY	-128.89	-128.89	anilide	*p*
4CM3	PTR1	KP2	PPY	-128.7	-128.7	H	*p*
3VS1	Hck	VSA	PPY	-128.02	-128.02	urea	*p*
5H0B	Hck	OOQ	PPY	-127.85	-127.85	OPh	*p*
5H0E	Hck	OOS	PPY	-127.81	-127.81	OPh	*p*
5H09	Hck	OOO	PPY	-127.63	-127.63	OPh	*p*
4CM4	PTR1	4NR	PPY	-127.15	-127.15	F	*p*
3VRY	Hck	B43	PPY	-125.32	-125.32	OPh	*p*
5H0G	Hck	OOU	PPY	-124.26	-124.26	OPh	*p*
3VS4	Hck	VSF	PPY	-124.03	-124.03	OPh	*p*
4YHF	Btk	4C9	PP	-123.98	-123.98	OPh	*p*
5TEH	RORg	S56	PPY	-122.82	-122.82	OPh	*p*
1QCF	Hck	PP1	PP	-122.62	-122.62	Me	*p*
3GEN	Btk	B43	PPY	-121.03	-121.03	OPh	*p*
4CMJ	PTR1	UIH	PPY	-120.74	-120.74	H	*p*
5P9I	Btk	1E8	PP	-120.41	-120.41	OPh	*p*
5ZJ6	Hck	VSE	PPY	-120.13	-120.13	OPh	*p*
4LUE	Hck	VSE	PPY	-119.59	-119.59	OPh	*p*
5P9J	Btk	8E8	PP	-119.57	-119.57	OPh	*p*
1QPE	Lck	PP2	PP	-119.4	-119.4	Cl	*p*
5YU9	Egfr (T790M)	1E8	PP	-117.54	-117.54	OPh	*p*
5FM2	Ret	PP1	PP	-117.04	-117.04	Me	*p*
2IVV	Ret	PP1	PP	-116.87	-116.87	Me	*p*
4IFG	CDPK1	1E8	PP	-116.22	-116.22	OPh	*p*
3GEQ	Src	PP2	PP	-112.99	-112.99	Cl	*p*
5FM3	Ret	PP1	PP	-111.74	-111.74	Me	*p*
2ZV9	Lyn	PP2	PP	-111.27	-111.27	Cl	*p*
3SXS	BMX	PP2	PP	-109.29	-109.29	Cl	*p*
3QLF	Src (L317I)	PD5	PP	-108.34	-108.34	anilide	*p*
2BRM	Chk1	DFZ	PPY	-106.44	-106.44	H	*p*
4D9T	Rsk2	0JG	PPY	-102.83	-102.83	Me	*p*
2BRO	Chk1	DF2	PPY	-98.54	-98.54	H	*p*
5T0P	Src	73A	PPY	-98.38	-98.38	Cl	*p*
4D9U	Rsk2	0JH	PPY	-97.04	-97.04	Me	*p*
5SWH	Src (V231C)	71D	PPY	-90.5	-90.5	Cl	*p*
4CME	PTR1	KTZ	PPY	-62.33	-62.33	H	*p*
6HJK	Aurora	G7W	PP	-57.19	-57.19	anilide	*p*
4CMC	PTR1	VS8	PPY	-52.29	-52.29	H	*p*
4CMA	PTR1	M2N	PPY	-52.11	-52.11	H	*p*
4CMG	PTR1	UHX	PPY	-50.16	-50.16	OMe	*p*
3EL8	Src	PD5	PP	-48.75	-48.75	anilide	*p*
4IH8	CDPK1	B43	PPY	-10.28	-10.28	OPh	*p*
6YAT	MST1	OJ5	PPY	-136.5	-136.5	Cl	*m*
7RUN	RET	7QU	PPY	-138.4	-138.4	NH2, Cl	*m, p*
7NB7	Mcl-1	U6N	PPY	-100.3	-100.3	Me, Cl	*o, m*
6TFU	EGFR T790M/V948R	N7K	PPY	-130.8	-130.8	amide	*m*
6TFV	EGFR T790M/V948R	N7Q	PPY	-131.8	-131.8	amide, ether	*m, p*
6TFY	EGFR T790M/V948R	N7Z	PPY	-128.9	-128.9	amide, ether	*m, p*
6TFZ	EGFR T790M/V948R	N7B	PPY	-126.1	-126.1	amide, ether	*m, p*
6TFW	EGFR T790M/V948R	N7W	PPY	-136.8	-136.8	amide, ether	*m, p*
6TG0	EGFR T790M/V948R	N78	PPY	-135.9	-135.9	amide, ether	*m, p*
6TG1	EGFR T790M/V948R	N82	PPY	-129.1	-129.1	amide, ether	*m, p*
6YZ4	MAP2K7	1E8	PP	-141	-141	OPh	*p*
6YG2	MAP2K7	8E8	PP	-122.7	-122.7	OPh	*p*
6L8L	C-Src	1E8	PP	-155.2	-155.2	OPh	*p*
7NG7	Src	UCW	PP	-123.4	-123.4	ether, amide	*m,p*

Supplementary Table 3: Partial kinome screen of **2** and **3** at 1 uM (in duplicate) against top 50 kinases that ibrutinib inhibits using Life Technologies SelectScreen® Kinase Profiling Services. The average % activity inhibited is listed for each compound against each kinase:

Monomethyl Ibrutinib Analog (2)		Dimethyl Ibrutinib Analog (3)	
**Kinase**	**% Inhibition**	**Kinase**	**% Inhibition**
ABL1	16	ABL1	0
ABL2 (Arg)	23	ABL2 (Arg)	0
BLK	107	BLK	100
BMX	100	BMX	94
BTK	110	BTK	100
CSK	81	CSK	15
CSNK1E (CK1 epsilon)	35	CSNK1E (CK1 epsilon)	9
EGFR (ErbB1) L858R	51	EGFR (ErbB1) L858R	2
EGFR (ErbB1) T790M L858R	25	EGFR (ErbB1) T790M L858R	6
EGFR (ErbB1) T790M	68	EGFR (ErbB1) T790M	6
EGFR (ErbB1)	89	EGFR (ErbB1)	0
ERBB2 (HER2)	71	ERBB2 (HER2)	2
ERBB4 (HER4)	101	ERBB4 (HER4)	93
FGFR1	57	FGFR1	-1
FGFR2	54	FGFR2	8
FGFR3	12	FGFR3	3
FGR	98	FGR	53
FLT3	44	FLT3	13
FLT4 (VEGFR3)	21	FLT4 (VEGFR3)	5
FRK (PTK5)	71	FRK (PTK5)	9
FYN	92	FYN	19
HCK	91	HCK	19
ITK	78	ITK	8
JAK3	65	JAK3	-7
KDR (VEGFR2)	23	KDR (VEGFR2)	5
KIT V559D	14	KIT V559D	1
KIT	15	KIT	1
LCK	100	LCK	74
LYN A	85	LYN A	13
LYN B	92	LYN B	28
PDGFRA (PDGFR alpha)	16	PDGFRA (PDGFR alpha)	3
PDGFRB (PDGFR beta)	6	PDGFRB (PDGFR beta)	0
PTK6 (Brk)	97	PTK6 (Brk)	69
RET	46	RET	5
SRC	95	SRC	43
SRMS (Srm)	95	SRMS (Srm)	22
TNK1	6	TNK1	2
TXK	99	TXK	94
YES1	98	YES1	45
EGFR (ErbB1) d746-750	94	EGFR (ErbB1) d746-750	23
ERN1	2	ERN1	2
MAP2K1 (MEK1) S218D S222D	12	MAP2K1 (MEK1) S218D S222D	4
MAP2K2 (MEK2)	20	MAP2K2 (MEK2)	2
MAP2K5 (MEK5)	32	MAP2K5 (MEK5)	10
RET M918T	47	RET M918T	6
RIPK2	95	RIPK2	29
TEC	100	TEC	91
TNK2 (ACK)	60	TNK2 (ACK)	4
TTK	2	TTK	-7
ZAK	2	ZAK	0

Supplementary Table 4: IC_{50} Values for **2** (Monomethyl ibrutinib analog, RH-1-111) and **3** (Dimethyl ibrutinib analog, ST-4-56) using Life Technologies SelectScreen® Kinase Profiling Services:

Project #	Compound Name	Kinase Tested	[ATP] Tested	IC50	Hillslope	R^2 Value	Conc of Compound	% Inhibition		Development Reaction Interference	Test Compound Interference		Z'	Kinase Part# / Lot#	Graph
			(µM)	(nM)			(nM)	Point 1	Point 2		Coumarin	Fluorescein			
SSBK13656_63179	Dimethyl Ibr	BMX	Km app	5.67	1.56	0.9998	10000	98	102	Pass	Pass	Pass	0.75	PV3371/1992619	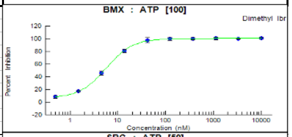
SSBK13656_63179	Dimethyl Ibr	BMX	Km app	5.67	1.56	0.9998	3330	99	100	Pass	Pass	Pass	0.75	PV3371/1992619	
SSBK13656_63179	Dimethyl Ibr	BMX	Km app	5.67	1.56	0.9998	1110	98	102	Pass	Pass	Pass	0.75	PV3371/1992619	
SSBK13656_63179	Dimethyl Ibr	BMX	Km app	5.67	1.56	0.9998	370	101	98	Pass	Pass	Pass	0.75	PV3371/1992619	
SSBK13656_63179	Dimethyl Ibr	BMX	Km app	5.67	1.56	0.9998	123	102	97	Pass	Pass	Pass	0.75	PV3371/1992619	
SSBK13656_63179	Dimethyl Ibr	BMX	Km app	5.67	1.56	0.9998	41.2	94	101	Pass	Pass	Pass	0.75	PV3371/1992619	
SSBK13656_63179	Dimethyl Ibr	BMX	Km app	5.67	1.56	0.9998	13.7	78	83	Pass	Pass	Pass	0.75	PV3371/1992619	
SSBK13656_63179	Dimethyl Ibr	BMX	Km app	5.67	1.56	0.9998	4.57	48	42	Pass	Pass	Pass	0.75	PV3371/1992619	
SSBK13656_63179	Dimethyl Ibr	BMX	Km app	5.67	1.56	0.9998	1.52	16	18	Pass	Pass	Pass	0.75	PV3371/1992619	
SSBK13656_63179	Dimethyl Ibr	BMX	Km app	5.67	1.56	0.9998	0.495	6	10	Pass	Pass	Pass	0.75	PV3371/1992619	
SSBK13656_63179	Dimethyl Ibr	SRC	Km app	1710	0.49	0.9729	10000	80	88	Pass	Pass	Pass	0.77	P3044/2289458	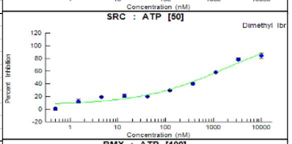
SSBK13656_63179	Dimethyl Ibr	SRC	Km app	1710	0.49	0.9729	3330	76	81	Pass	Pass	Pass	0.77	P3044/2289458	
SSBK13656_63179	Dimethyl Ibr	SRC	Km app	1710	0.49	0.9729	1110	57	58	Pass	Pass	Pass	0.77	P3044/2289458	
SSBK13656_63179	Dimethyl Ibr	SRC	Km app	1710	0.49	0.9729	370	38	42	Pass	Pass	Pass	0.77	P3044/2289458	
SSBK13656_63179	Dimethyl Ibr	SRC	Km app	1710	0.49	0.9729	123	30	28	Pass	Pass	Pass	0.77	P3044/2289458	
SSBK13656_63179	Dimethyl Ibr	SRC	Km app	1710	0.49	0.9729	41.2	19	20	Pass	Pass	Pass	0.77	P3044/2289458	
SSBK13656_63179	Dimethyl Ibr	SRC	Km app	1710	0.49	0.9729	13.7	23	18	Pass	Pass	Pass	0.77	P3044/2289458	
SSBK13656_63179	Dimethyl Ibr	SRC	Km app	1710	0.49	0.9729	4.57	19	20	Pass	Pass	Pass	0.77	P3044/2289458	
SSBK13656_63179	Dimethyl Ibr	SRC	Km app	1710	0.49	0.9729	1.52	15	9	Pass	Pass	Pass	0.77	P3044/2289458	
SSBK13656_63179	Dimethyl Ibr	SRC	Km app	1710	0.49	0.9729	0.495	2	-2	Pass	Pass	Pass	0.77	P3044/2289458	
SSBK13656_63179	Monomethyl Ibr	BMX	Km app	1.18	1.13	0.9912	10000	102	98	Pass	Pass	Pass	0.75	PV3371/1992619	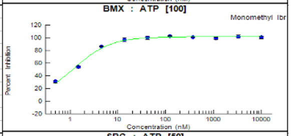
SSBK13656_63179	Monomethyl Ibr	BMX	Km app	1.18	1.13	0.9912	3330	99	104	Pass	Pass	Pass	0.75	PV3371/1992619	
SSBK13656_63179	Monomethyl Ibr	BMX	Km app	1.18	1.13	0.9912	1110	101	97	Pass	Pass	Pass	0.75	PV3371/1992619	
SSBK13656_63179	Monomethyl Ibr	BMX	Km app	1.18	1.13	0.9912	370	100	100	Pass	Pass	Pass	0.75	PV3371/1992619	
SSBK13656_63179	Monomethyl Ibr	BMX	Km app	1.18	1.13	0.9912	123	101	103	Pass	Pass	Pass	0.75	PV3371/1992619	
SSBK13656_63179	Monomethyl Ibr	BMX	Km app	1.18	1.13	0.9912	41.2	102	97	Pass	Pass	Pass	0.75	PV3371/1992619	
SSBK13656_63179	Monomethyl Ibr	BMX	Km app	1.18	1.13	0.9912	13.7	99	94	Pass	Pass	Pass	0.75	PV3371/1992619	
SSBK13656_63179	Monomethyl Ibr	BMX	Km app	1.18	1.13	0.9912	4.57	86	86	Pass	Pass	Pass	0.75	PV3371/1992619	
SSBK13656_63179	Monomethyl Ibr	BMX	Km app	1.18	1.13	0.9912	1.52	52	55	Pass	Pass	Pass	0.75	PV3371/1992619	
SSBK13656_63179	Monomethyl Ibr	BMX	Km app	1.18	1.13	0.9912	0.495	29	33	Pass	Pass	Pass	0.75	PV3371/1992619	
SSBK13656_63179	Monomethyl Ibr	SRC	Km app	33.5	0.99	0.9992	10000	98	101	Pass	Pass	Pass	0.77	P3044/2289458	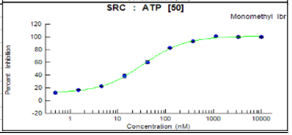
SSBK13656_63179	Monomethyl Ibr	SRC	Km app	33.5	0.99	0.9992	3330	100	98	Pass	Pass	Pass	0.77	P3044/2289458	
SSBK13656_63179	Monomethyl Ibr	SRC	Km app	33.5	0.99	0.9992	1110	100	101	Pass	Pass	Pass	0.77	P3044/2289458	
SSBK13656_63179	Monomethyl Ibr	SRC	Km app	33.5	0.99	0.9992	370	93	92	Pass	Pass	Pass	0.77	P3044/2289458	
SSBK13656_63179	Monomethyl Ibr	SRC	Km app	33.5	0.99	0.9992	123	82	82	Pass	Pass	Pass	0.77	P3044/2289458	
SSBK13656_63179	Monomethyl Ibr	SRC	Km app	33.5	0.99	0.9992	41.2	62	58	Pass	Pass	Pass	0.77	P3044/2289458	
SSBK13656_63179	Monomethyl Ibr	SRC	Km app	33.5	0.99	0.9992	13.7	41	35	Pass	Pass	Pass	0.77	P3044/2289458	
SSBK13656_63179	Monomethyl Ibr	SRC	Km app	33.5	0.99	0.9992	4.57	22	22	Pass	Pass	Pass	0.77	P3044/2289458	
SSBK13656_63179	Monomethyl Ibr	SRC	Km app	33.5	0.99	0.9992	1.52	17	15	Pass	Pass	Pass	0.77	P3044/2289458	
SSBK13656_63179	Monomethyl Ibr	SRC	Km app	33.5	0.99	0.9992	0.495	10	13	Pass	Pass	Pass	0.77	P3044/2289458	

Project #	Compound Name	Kinase Tested	[ATP] Tested	IC50	Hillslope	R^2 Value	Conc of Compound	% Inhibition		Development Reaction Interference	Test Compound Interference		Z'	Kinase Part# / Lot#	Graph
			(µM)	(nM)			(nM)	Point 1	Point 2		Coumarin	Fluorescein			
SSBK13105_58093	RH-1-111	BLK	Km app	1.36	1.06	0.9921	10000	97	99	Pass	Pass	Pass	0.71	PV3683/1823936	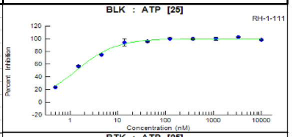
SSBK13105_58093	RH-1-111	BLK	Km app	1.36	1.06	0.9921	3330	101	103	Pass	Pass	Pass	0.71	PV3683/1823936	
SSBK13105_58093	RH-1-111	BLK	Km app	1.36	1.06	0.9921	1110	97	102	Pass	Pass	Pass	0.71	PV3683/1823936	
SSBK13105_58093	RH-1-111	BLK	Km app	1.36	1.06	0.9921	370	98	101	Pass	Pass	Pass	0.71	PV3683/1823936	
SSBK13105_58093	RH-1-111	BLK	Km app	1.36	1.06	0.9921	123	101	99	Pass	Pass	Pass	0.71	PV3683/1823936	
SSBK13105_58093	RH-1-111	BLK	Km app	1.36	1.06	0.9921	41.2	94	96	Pass	Pass	Pass	0.71	PV3683/1823936	
SSBK13105_58093	RH-1-111	BLK	Km app	1.36	1.06	0.9921	13.7	99	88	Pass	Pass	Pass	0.71	PV3683/1823936	
SSBK13105_58093	RH-1-111	BLK	Km app	1.36	1.06	0.9921	4.57	74	75	Pass	Pass	Pass	0.71	PV3683/1823936	
SSBK13105_58093	RH-1-111	BLK	Km app	1.36	1.06	0.9921	1.52	55	58	Pass	Pass	Pass	0.71	PV3683/1823936	
SSBK13105_58093	RH-1-111	BLK	Km app	1.36	1.06	0.9921	0.495	23	23	Pass	Pass	Pass	0.71	PV3683/1823936	
SSBK13105_58093	RH-1-111	BTK	Km app	0.936	1.84	0.9962	10000	123	96	Pass	Pass	Pass	0.80	PV3363/1974713	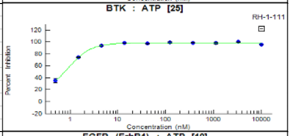
SSBK13105_58093	RH-1-111	BTK	Km app	0.936	1.84	0.9962	3330	100	101	Pass	Pass	Pass	0.80	PV3363/1974713	
SSBK13105_58093	RH-1-111	BTK	Km app	0.936	1.84	0.9962	1110	100	97	Pass	Pass	Pass	0.80	PV3363/1974713	
SSBK13105_58093	RH-1-111	BTK	Km app	0.936	1.84	0.9962	370	100	98	Pass	Pass	Pass	0.80	PV3363/1974713	
SSBK13105_58093	RH-1-111	BTK	Km app	0.936	1.84	0.9962	123	98	100	Pass	Pass	Pass	0.80	PV3363/1974713	
SSBK13105_58093	RH-1-111	BTK	Km app	0.936	1.84	0.9962	41.2	97	98	Pass	Pass	Pass	0.80	PV3363/1974713	
SSBK13105_58093	RH-1-111	BTK	Km app	0.936	1.84	0.9962	13.7	99	98	Pass	Pass	Pass	0.80	PV3363/1974713	
SSBK13105_58093	RH-1-111	BTK	Km app	0.936	1.84	0.9962	4.57	93	95	Pass	Pass	Pass	0.80	PV3363/1974713	
SSBK13105_58093	RH-1-111	BTK	Km app	0.936	1.84	0.9962	1.52	73	75	Pass	Pass	Pass	0.80	PV3363/1974713	
SSBK13105_58093	RH-1-111	BTK	Km app	0.936	1.84	0.9962	0.495	32	39	Pass	Pass	Pass	0.80	PV3363/1974713	
SSBK13105_58093	RH-1-111	EGFR (ErbB1)	Km app	197	1.08	0.9981	10000	97	95	Pass	Pass	Pass	0.83	PV3872/1729964	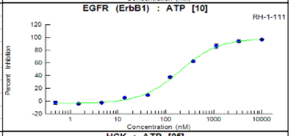
SSBK13105_58093	RH-1-111	EGFR (ErbB1)	Km app	197	1.08	0.9981	3330	92	96	Pass	Pass	Pass	0.83	PV3872/1729964	
SSBK13105_58093	RH-1-111	EGFR (ErbB1)	Km app	197	1.08	0.9981	1110	89	82	Pass	Pass	Pass	0.83	PV3872/1729964	
SSBK13105_58093	RH-1-111	EGFR (ErbB1)	Km app	197	1.08	0.9981	370	60	63	Pass	Pass	Pass	0.83	PV3872/1729964	
SSBK13105_58093	RH-1-111	EGFR (ErbB1)	Km app	197	1.08	0.9981	123	38	35	Pass	Pass	Pass	0.83	PV3872/1729964	
SSBK13105_58093	RH-1-111	EGFR (ErbB1)	Km app	197	1.08	0.9981	41.2	10	8	Pass	Pass	Pass	0.83	PV3872/1729964	
SSBK13105_58093	RH-1-111	EGFR (ErbB1)	Km app	197	1.08	0.9981	13.7	4	5	Pass	Pass	Pass	0.83	PV3872/1729964	
SSBK13105_58093	RH-1-111	EGFR (ErbB1)	Km app	197	1.08	0.9981	4.57	-2	-4	Pass	Pass	Pass	0.83	PV3872/1729964	
SSBK13105_58093	RH-1-111	EGFR (ErbB1)	Km app	197	1.08	0.9981	1.52	-4	-6	Pass	Pass	Pass	0.83	PV3872/1729964	
SSBK13105_58093	RH-1-111	EGFR (ErbB1)	Km app	197	1.08	0.9981	0.495	-6	-1	Pass	Pass	Pass	0.83	PV3872/1729964	
SSBK13105_58093	RH-1-111	HCK	Km app	29.5	0.70	0.9988	10000	98	100	Pass	Pass	Pass	0.87	PV6128/2071130	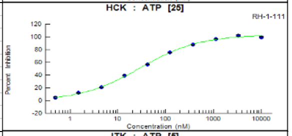
SSBK13105_58093	RH-1-111	HCK	Km app	29.5	0.70	0.9988	3330	100	103	Pass	Pass	Pass	0.87	PV6128/2071130	
SSBK13105_58093	RH-1-111	HCK	Km app	29.5	0.70	0.9988	1110	97	96	Pass	Pass	Pass	0.87	PV6128/2071130	
SSBK13105_58093	RH-1-111	HCK	Km app	29.5	0.70	0.9988	370	87	88	Pass	Pass	Pass	0.87	PV6128/2071130	
SSBK13105_58093	RH-1-111	HCK	Km app	29.5	0.70	0.9988	123	75	75	Pass	Pass	Pass	0.87	PV6128/2071130	
SSBK13105_58093	RH-1-111	HCK	Km app	29.5	0.70	0.9988	41.2	57	56	Pass	Pass	Pass	0.87	PV6128/2071130	
SSBK13105_58093	RH-1-111	HCK	Km app	29.5	0.70	0.9988	13.7	38	40	Pass	Pass	Pass	0.87	PV6128/2071130	
SSBK13105_58093	RH-1-111	HCK	Km app	29.5	0.70	0.9988	4.57	21	20	Pass	Pass	Pass	0.87	PV6128/2071130	
SSBK13105_58093	RH-1-111	HCK	Km app	29.5	0.70	0.9988	1.52	11	12	Pass	Pass	Pass	0.87	PV6128/2071130	
SSBK13105_58093	RH-1-111	HCK	Km app	29.5	0.70	0.9988	0.495	6	3	Pass	Pass	Pass	0.87	PV6128/2071130	
SSBK13105_58093	RH-1-111	ITK	Km app	344	1.16	0.9997	10000	96	99	Pass	Pass	Pass	0.76	PV3875/1860356	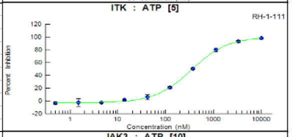
SSBK13105_58093	RH-1-111	ITK	Km app	344	1.16	0.9997	3330	91	94	Pass	Pass	Pass	0.76	PV3875/1860356	
SSBK13105_58093	RH-1-111	ITK	Km app	344	1.16	0.9997	1110	76	83	Pass	Pass	Pass	0.76	PV3875/1860356	
SSBK13105_58093	RH-1-111	ITK	Km app	344	1.16	0.9997	370	49	51	Pass	Pass	Pass	0.76	PV3875/1860356	
SSBK13105_58093	RH-1-111	ITK	Km app	344	1.16	0.9997	123	18	22	Pass	Pass	Pass	0.76	PV3875/1860356	
SSBK13105_58093	RH-1-111	ITK	Km app	344	1.16	0.9997	41.2	2	9	Pass	Pass	Pass	0.76	PV3875/1860356	
SSBK13105_58093	RH-1-111	ITK	Km app	344	1.16	0.9997	13.7	0	2	Pass	Pass	Pass	0.76	PV3875/1860356	
SSBK13105_58093	RH-1-111	ITK	Km app	344	1.16	0.9997	4.57	-4	-3	Pass	Pass	Pass	0.76	PV3875/1860356	
SSBK13105_58093	RH-1-111	ITK	Km app	344	1.16	0.9997	1.52	3	-10	Pass	Pass	Pass	0.76	PV3875/1860356	
SSBK13105_58093	RH-1-111	ITK	Km app	344	1.16	0.9997	0.495	-6	-2	Pass	Pass	Pass	0.76	PV3875/1860356	
SSBK13105_58093	RH-1-111	JAK3	Km app	874	1.09	0.9965	10000	98	93	Pass	Pass	Pass	0.85	PV3855/1857644	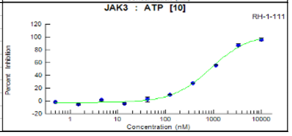
SSBK13105_58093	RH-1-111	JAK3	Km app	874	1.09	0.9965	3330	89	85	Pass	Pass	Pass	0.85	PV3855/1857644	
SSBK13105_58093	RH-1-111	JAK3	Km app	874	1.09	0.9965	1110	55	56	Pass	Pass	Pass	0.85	PV3855/1857644	
SSBK13105_58093	RH-1-111	JAK3	Km app	874	1.09	0.9965	370	28	27	Pass	Pass	Pass	0.85	PV3855/1857644	
SSBK13105_58093	RH-1-111	JAK3	Km app	874	1.09	0.9965	123	9	10	Pass	Pass	Pass	0.85	PV3855/1857644	
SSBK13105_58093	RH-1-111	JAK3	Km app	874	1.09	0.9965	41.2	6	-2	Pass	Pass	Pass	0.85	PV3855/1857644	
SSBK13105_58093	RH-1-111	JAK3	Km app	874	1.09	0.9965	13.7	-5	-3	Pass	Pass	Pass	0.85	PV3855/1857644	
SSBK13105_58093	RH-1-111	JAK3	Km app	874	1.09	0.9965	4.57	2	0	Pass	Pass	Pass	0.85	PV3855/1857644	
SSBK13105_58093	RH-1-111	JAK3	Km app	874	1.09	0.9965	1.52	-7	-5	Pass	Pass	Pass	0.85	PV3855/1857644	
SSBK13105_58093	RH-1-111	JAK3	Km app	874	1.09	0.9965	0.495	-1	-4	Pass	Pass	Pass	0.85	PV3855/1857644	

SSBK13105_58093	STT-4-56	BLK	Km app	35.6	1.08	0.9992	10000	101	101	Pass	Pass	Pass	0.71	PV3683/1823936
SSBK13105_58093	STT-4-56	BLK	Km app	35.6	1.08	0.9992	3330	101	102	Pass	Pass	Pass	0.71	PV3683/1823936
SSBK13105_58093	STT-4-56	BLK	Km app	35.6	1.08	0.9992	1110	101	101	Pass	Pass	Pass	0.71	PV3683/1823936
SSBK13105_58093	STT-4-56	BLK	Km app	35.6	1.08	0.9992	370	98	96	Pass	Pass	Pass	0.71	PV3683/1823936
SSBK13105_58093	STT-4-56	BLK	Km app	35.6	1.08	0.9992	123	77	82	Pass	Pass	Pass	0.71	PV3683/1823936
SSBK13105_58093	STT-4-56	BLK	Km app	35.6	1.08	0.9992	41.2	56	55	Pass	Pass	Pass	0.71	PV3683/1823936
SSBK13105_58093	STT-4-56	BLK	Km app	35.6	1.08	0.9992	13.7	30	24	Pass	Pass	Pass	0.71	PV3683/1823936
SSBK13105_58093	STT-4-56	BLK	Km app	35.6	1.08	0.9992	4.57	13	7	Pass	Pass	Pass	0.71	PV3683/1823936
SSBK13105_58093	STT-4-56	BLK	Km app	35.6	1.08	0.9992	1.52	5	-3	Pass	Pass	Pass	0.71	PV3683/1823936
SSBK13105_58093	STT-4-56	BLK	Km app	35.6	1.08	0.9992	0.495	-5	8	Pass	Pass	Pass	0.71	PV3683/1823936
SSBK13105_58093	STT-4-56	BTK	Km app	12.8	1.25	0.9974	10000	98	99	Pass	Pass	Pass	0.80	PV3363/1974713
SSBK13105_58093	STT-4-56	BTK	Km app	12.8	1.25	0.9974	3330	98	97	Pass	Pass	Pass	0.80	PV3363/1974713
SSBK13105_58093	STT-4-56	BTK	Km app	12.8	1.25	0.9974	1110	100	102	Pass	Pass	Pass	0.80	PV3363/1974713
SSBK13105_58093	STT-4-56	BTK	Km app	12.8	1.25	0.9974	370	98	98	Pass	Pass	Pass	0.80	PV3363/1974713
SSBK13105_58093	STT-4-56	BTK	Km app	12.8	1.25	0.9974	123	97	96	Pass	Pass	Pass	0.80	PV3363/1974713
SSBK13105_58093	STT-4-56	BTK	Km app	12.8	1.25	0.9974	41.2	84	80	Pass	Pass	Pass	0.80	PV3363/1974713
SSBK13105_58093	STT-4-56	BTK	Km app	12.8	1.25	0.9974	13.7	49	49	Pass	Pass	Pass	0.80	PV3363/1974713
SSBK13105_58093	STT-4-56	BTK	Km app	12.8	1.25	0.9974	4.57	23	22	Pass	Pass	Pass	0.80	PV3363/1974713
SSBK13105_58093	STT-4-56	BTK	Km app	12.8	1.25	0.9974	1.52	11	5	Pass	Pass	Pass	0.80	PV3363/1974713
SSBK13105_58093	STT-4-56	BTK	Km app	12.8	1.25	0.9974	0.495	-4	1	Pass	Pass	Pass	0.80	PV3363/1974713
SSBK13105_58093	STT-4-56	EGFR (ErbB1)	Km app	>10000	0.96	0.9911	10000	41	45	Pass	Pass	Pass	0.83	PV3872/1729964
SSBK13105_58093	STT-4-56	EGFR (ErbB1)	Km app	>10000	0.96	0.9911	3330	20	22	Pass	Pass	Pass	0.83	PV3872/1729964
SSBK13105_58093	STT-4-56	EGFR (ErbB1)	Km app	>10000	0.96	0.9911	1110	10	5	Pass	Pass	Pass	0.83	PV3872/1729964
SSBK13105_58093	STT-4-56	EGFR (ErbB1)	Km app	>10000	0.96	0.9911	370	5	1	Pass	Pass	Pass	0.83	PV3872/1729964
SSBK13105_58093	STT-4-56	EGFR (ErbB1)	Km app	>10000	0.96	0.9911	123	1	-2	Pass	Pass	Pass	0.83	PV3872/1729964
SSBK13105_58093	STT-4-56	EGFR (ErbB1)	Km app	>10000	0.96	0.9911	41.2	-6	-3	Pass	Pass	Pass	0.83	PV3872/1729964
SSBK13105_58093	STT-4-56	EGFR (ErbB1)	Km app	>10000	0.96	0.9911	13.7	-1	0	Pass	Pass	Pass	0.83	PV3872/1729964
SSBK13105_58093	STT-4-56	EGFR (ErbB1)	Km app	>10000	0.96	0.9911	4.57	-2	1	Pass	Pass	Pass	0.83	PV3872/1729964
SSBK13105_58093	STT-4-56	EGFR (ErbB1)	Km app	>10000	0.96	0.9911	1.52	-1	-2	Pass	Pass	Pass	0.83	PV3872/1729964
SSBK13105_58093	STT-4-56	EGFR (ErbB1)	Km app	>10000	0.96	0.9911	0.495	-4	3	Pass	Pass	Pass	0.83	PV3872/1729964
SSBK13105_58093	STT-4-56	HCK	Km app	2310	1.00	0.9992	10000	62	65	Pass	Pass	Pass	0.87	PV6128/2071130
SSBK13105_58093	STT-4-56	HCK	Km app	2310	1.00	0.9992	3330	48	49	Pass	Pass	Pass	0.87	PV6128/2071130
SSBK13105_58093	STT-4-56	HCK	Km app	2310	1.00	0.9992	1110	32	29	Pass	Pass	Pass	0.87	PV6128/2071130
SSBK13105_58093	STT-4-56	HCK	Km app	2310	1.00	0.9992	370	19	15	Pass	Pass	Pass	0.87	PV6128/2071130
SSBK13105_58093	STT-4-56	HCK	Km app	2310	1.00	0.9992	123	10	15	Pass	Pass	Pass	0.87	PV6128/2071130
SSBK13105_58093	STT-4-56	HCK	Km app	2310	1.00	0.9992	41.2	9	8	Pass	Pass	Pass	0.87	PV6128/2071130
SSBK13105_58093	STT-4-56	HCK	Km app	2310	1.00	0.9992	13.7	6	10	Pass	Pass	Pass	0.87	PV6128/2071130
SSBK13105_58093	STT-4-56	HCK	Km app	2310	1.00	0.9992	4.57	10	5	Pass	Pass	Pass	0.87	PV6128/2071130
SSBK13105_58093	STT-4-56	HCK	Km app	2310	1.00	0.9992	1.52	9	7	Pass	Pass	Pass	0.87	PV6128/2071130
SSBK13105_58093	STT-4-56	HCK	Km app	2310	1.00	0.9992	0.495	10	7	Pass	Pass	Pass	0.87	PV6128/2071130
SSBK13105_58093	STT-4-56	ITK	Km app	>10000	1.28	0.9661	10000	51	47	Pass	Pass	Pass	0.76	PV3875/1860356
SSBK13105_58093	STT-4-56	ITK	Km app	>10000	1.28	0.9661	3330	13	16	Pass	Pass	Pass	0.76	PV3875/1860356
SSBK13105_58093	STT-4-56	ITK	Km app	>10000	1.28	0.9661	1110	8	4	Pass	Pass	Pass	0.76	PV3875/1860356
SSBK13105_58093	STT-4-56	ITK	Km app	>10000	1.28	0.9661	370	5	5	Pass	Pass	Pass	0.76	PV3875/1860356
SSBK13105_58093	STT-4-56	ITK	Km app	>10000	1.28	0.9661	123	-2	0	Pass	Pass	Pass	0.76	PV3875/1860356
SSBK13105_58093	STT-4-56	ITK	Km app	>10000	1.28	0.9661	41.2	-4	-2	Pass	Pass	Pass	0.76	PV3875/1860356
SSBK13105_58093	STT-4-56	ITK	Km app	>10000	1.28	0.9661	13.7	-4	1	Pass	Pass	Pass	0.76	PV3875/1860356
SSBK13105_58093	STT-4-56	ITK	Km app	>10000	1.28	0.9661	4.57	-1	-1	Pass	Pass	Pass	0.76	PV3875/1860356
SSBK13105_58093	STT-4-56	ITK	Km app	>10000	1.28	0.9661	1.52	-11	-5	Pass	Pass	Pass	0.76	PV3875/1860356
SSBK13105_58093	STT-4-56	ITK	Km app	>10000	1.28	0.9661	0.495	-12	7	Pass	Pass	Pass	0.76	PV3875/1860356
SSBK13105_58093	STT-4-56	JAK3	Km app	>10000	0.86	0.9863	10000	32	32	Pass	Pass	Pass	0.85	PV3855/1857644
SSBK13105_58093	STT-4-56	JAK3	Km app	>10000	0.86	0.9863	3330	17	14	Pass	Pass	Pass	0.85	PV3855/1857644
SSBK13105_58093	STT-4-56	JAK3	Km app	>10000	0.86	0.9863	1110	6	11	Pass	Pass	Pass	0.85	PV3855/1857644
SSBK13105_58093	STT-4-56	JAK3	Km app	>10000	0.86	0.9863	370	3	0	Pass	Pass	Pass	0.85	PV3855/1857644
SSBK13105_58093	STT-4-56	JAK3	Km app	>10000	0.86	0.9863	123	2	4	Pass	Pass	Pass	0.85	PV3855/1857644
SSBK13105_58093	STT-4-56	JAK3	Km app	>10000	0.86	0.9863	41.2	1	3	Pass	Pass	Pass	0.85	PV3855/1857644
SSBK13105_58093	STT-4-56	JAK3	Km app	>10000	0.86	0.9863	13.7	1	1	Pass	Pass	Pass	0.85	PV3855/1857644
SSBK13105_58093	STT-4-56	JAK3	Km app	>10000	0.86	0.9863	4.57	2	-1	Pass	Pass	Pass	0.85	PV3855/1857644
SSBK13105_58093	STT-4-56	JAK3	Km app	>10000	0.86	0.9863	1.52	-1	-1	Pass	Pass	Pass	0.85	PV3855/1857644
SSBK13105_58093	STT-4-56	JAK3	Km app	>10000	0.86	0.9863	0.495	4	1	Pass	Pass	Pass	0.85	PV3855/1857644

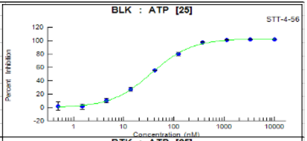

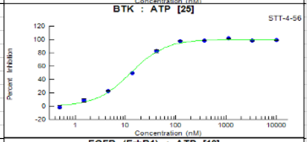

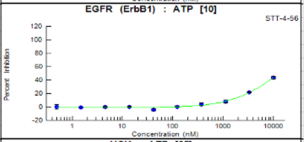

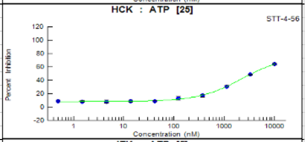

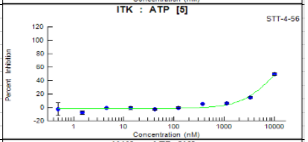

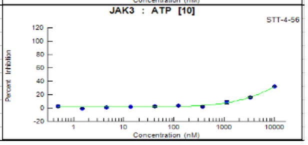

Supplementary Table 5: AssayQuant Technologies k_{inact}/K_i Profiling for **2** (monomethyl ibrutinib analog), and **3** (dimethyl ibrutinib analog) using PhosphoSens® CSox-based Kinetic Assay Format:

Compound	Enzyme Target	Enzyme conc. (nM)	Date	ATP (µM)	Sensor (µM)	1- or 2-step	kinact / KI (M-1sec-1)		kinact (sec-1)		KI app (µM)		R^2
							Fitted value	Std. error	Fitted value	Std. error	Fitted value	Std. error	
Monomethyl Analog	BLK	2.5	6/24/22	1000	15	Two step	316,000	8,000	0.0034	0.0002	0.0106	0.0007	0.98
Dimethyl Analog	BLK	2.5	6/24/22	1000	15	Two step	35,500	1,400	0.0021	0.0001	0.0583	0.0043	0.97
Ibrutinib	BLK	2.5	6/24/22	1000	15	Two step	710,000	18,000	0.0034	0.0002	0.0047	0.0003	0.98
Monomethyl Analog	BMX	5.0	6/24/22	1000	15	Two step	80,800	2,300	0.0065	0.0007	0.0806	0.0103	0.98
Dimethyl Analog	BMX	5.0	6/24/22	1000	15	Two step	64,800	2,900	0.0053	0.0006	0.0818	0.0114	0.95
Ibrutinib	HCK	2.5	6/27/22	1000	15	Two step	22,100	1,000	0.0018	0.0001	0.0806	0.0058	0.94
Monomethyl Analog	HCK	2.5	6/27/22	1000	15	Two step	1,730	150	0.0009	0.0001	0.5270	0.0670	0.94
Dimethyl Analog	HCK	2.5	6/27/22	1000	15	Two step	5,960	2,180	0.0001	0.0000	0.0183	0.0066	0.92
Monomethyl Analog	ITK	15.0	6/27/22	1000	15	Two step	4,120	2,880	0.0001	0.0000	0.0359	0.0258	0.80
Dimethyl Analog	ITK	15.0	6/27/22	1000	15	Two step	2,800	890	0.0005	0.0001	0.1620	0.0620	0.76

Compound	Enzyme Target	Enzyme conc. (nM)	Date	ATP (µM)	Sensor (µM)	1- or 2-step	kinact / KI (M-1sec-1)		kinact (sec-1)		KI app (µM)		R^2
							Fitted value	Std. error	Fitted value	Std. error	Fitted value	Std. error	
Dimethyl Analog	BTK	0.6	2/8/22	1000	15	One step	31,600	800	N/A	N/A	N/A	N/A	0.9866
Monomethyl Analog	BTK	0.6	2/8/22	1000	15	One step	280,000	5,000	N/A	N/A	N/A	N/A	0.9900
Ibrutinib (Control)	BTK	0.6	2/8/22	1000	15	One step	328,000	9,000	N/A	N/A	N/A	N/A	0.9767

Western Blot Experiments: Jeko-1 cells (500,000 per condition) were treated with serially diluted ibrutinib (10 uM to 0.1 uM), **2** (10 uM to 0.01 uM), **3** (10 uM to 0.01 uM), or DMSO for 2 h. After 2h, cells were washed with cold phosphate-buffered solution (PBS) and lysed in radioimmunoprecipitation assay (RIPA) lysis buffer supplemented with a 1x protease/phosphatase inhibitor cocktail (Cell Signaling Technology, #5872). The protein concentration for each extract was measured using the bicinchoninic acid (BCA) protein assay reagent (Thermo Scientific). Western blot analyses were conducted using a Simple Western Automated Processor according to the manufacturer's protocol. Samples were incubated with phospho-BTK (Y223; Cell Signaling Technology, #5082 1:50), and total BTK (Cell Signaling Technology, #8547, 1:200) antibodies for protein quantification.

Full Western Blot Gel for PhosphoBTK:

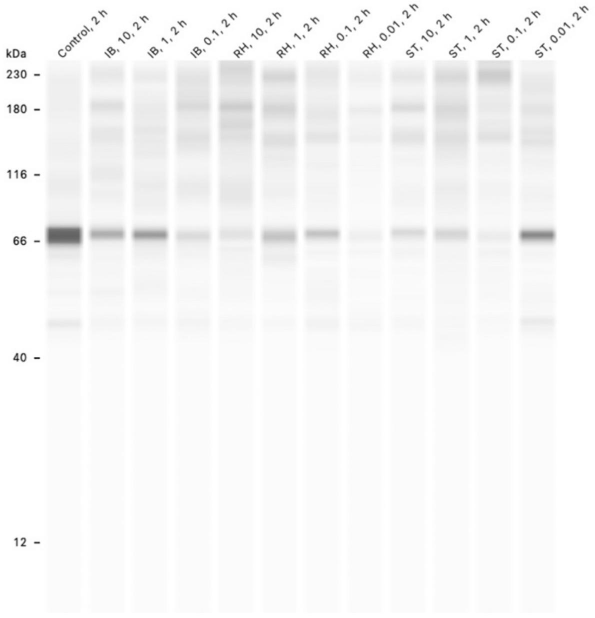

Full Western Blot Gel for Total BTK:

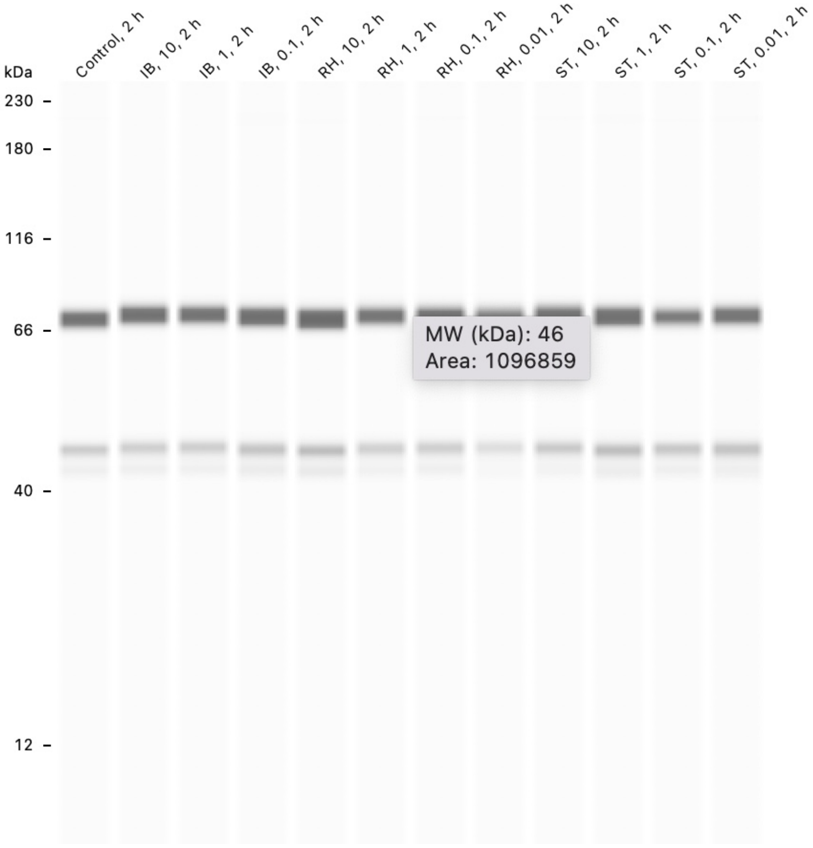

NanoBRET Experiments: HEK293 cells were transfected with the BTK-NanoLuc fusion vector (Promega, #N2501) according to the manufacturer's protocol. After transfection, cells were incubated with serially diluted concentrations of **2, 3,** Ibrutinib, or Dasatinib and the NanoBRET tracer reagent (0.25 uM prepared in tracer dilution buffer, Promega, #N2501) for 30 minutes. After an additional 30, 60, or 90 minutes the NanoBRET complete substrate plus inhibitor solution (3X solution in Opti-MEM I reduced serum medium, no phenol red, Promega, #N2501) was added to each well. After 5-10 minutes, the BRET ratio was measured using BMG Labtech CLARIOstar with preloaded filters for donor 450nm/80nm BP (band pass) and acceptor 610nm LP (long pass).

NanoBRET IC_{50} Values (nM):

Time	**30 min**	**60 min**	**90 min**	**120 min**
**2**	210.8	154.1	118.3	108.9
Ibrutinib	73.5	54.3	43.8	44.2
Dasatinib	114.2	68.6	53.5	47.9
**3**	805.8	473.6	340.8	314.8

Error Analysis for NanoBRET IC_{50} Values (nM):

Time	**30 min**	**60 min**	**90 min**	**120 min**
**2**	+/- 22.89	+/- 12.26	+/- 14.62	+/- 16.22
Ibrutinib	+/- 12.57	+/- 9.1	+/- 8.455	+/- 6.35
Dasatinib	+/- 26.43	+/- 10.77	+/- 9.796	+/- 6.35
**3**	+/- 83.56	+/- 48.81	+/- 38.85	+/- 26.84

Additional Graphs for Time Dependence of NanoBRET IC_{50} Values (nM) for **3** (leftmost graph), **2**, Ibrutinib, and Dasatinib:

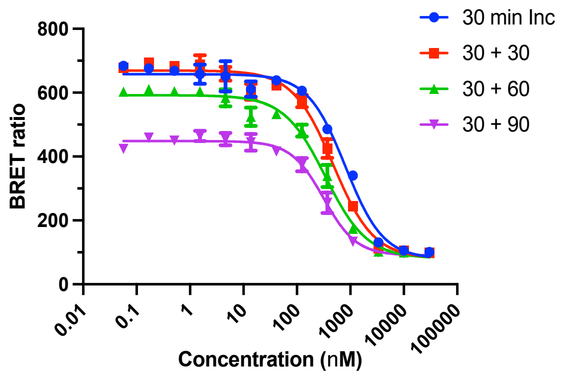

	30 min Inc	30 + 30	30 + 60	30 + 90
IC50	805.8	473.6	340.8	314.8
HillSlope	-1.230	-1.148	-1.118	-1.419

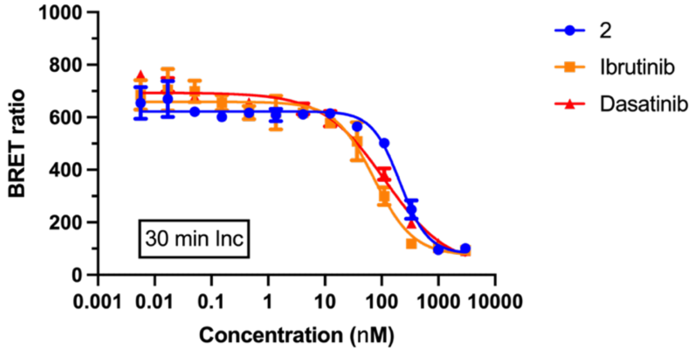

	2	Ibrutinib	Dasatinib
IC50	210.8	73.54	114.2
HillSlope	-1.761	-1.254	-0.7910

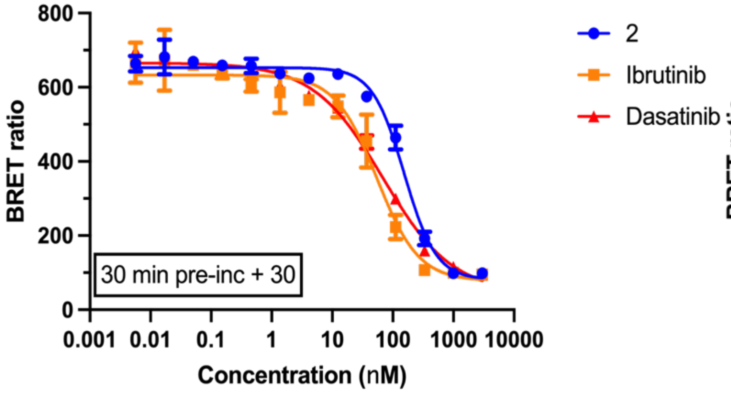

	2	Ibrutinib	Dasatinib
IC50	154.1	54.26	68.61
HillSlope	-1.651	-1.256	-0.7557

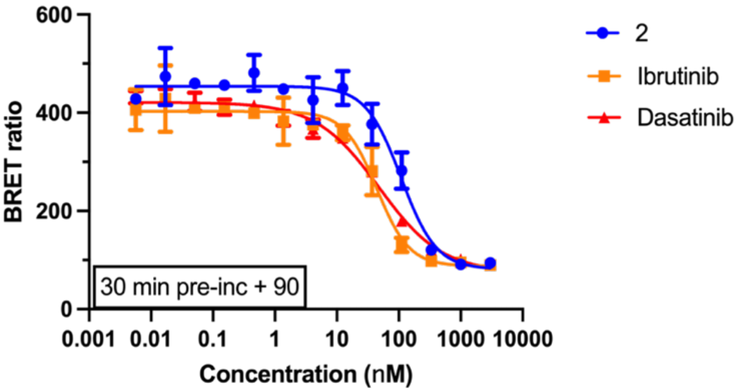

	2	Ibrutinib	Dasatinib
IC50	108.9	44.16	47.90
HillSlope	-1.532	-1.656	-0.8521

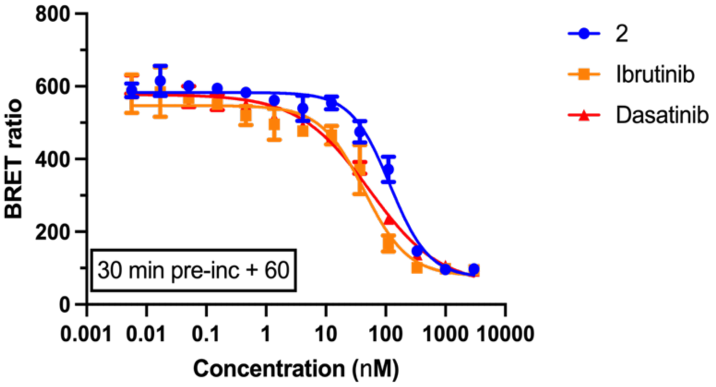

	2	Ibrutinib	Dasatinib
IC50	118.3	43.81	53.50
HillSlope	-1.334	-1.184	-0.7344

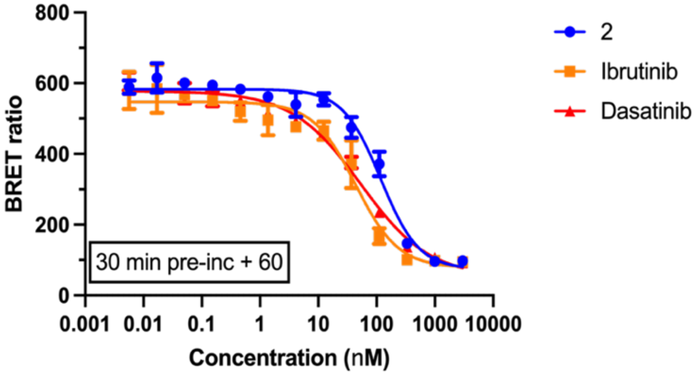

	2	Ibrutinib	Dasatinib
IC50	118.3	43.81	53.50
HillSlope	-1.334	-1.184	-0.7344
